# Rhizospheric miRNAs affect the plant microbiota

**DOI:** 10.1093/ismeco/ycae120

**Published:** 2024-10-12

**Authors:** Harriet Middleton, Jessica Ann Dozois, Cécile Monard, Virginie Daburon, Emmanuel Clostres, Julien Tremblay, Jean-Philippe Combier, Étienne Yergeau, Abdelhak El Amrani

**Affiliations:** Écosystèmes, Biodiversité, Évolution (ECOBIO), Unité mixte de recherche (UMR) 6553, Centre national de la recherche scientifique (CNRS) - Université de Rennes, Campus Beaulieu, 263 Avenue du Général Leclerc, Rennes, 35042, France; Institut National de la Recherche Scientifique, Centre Armand-Frappier Santé Biotechnologie, 531 boulevard des Prairies, Laval, Québec, H7V 1B7, Canada; Institut National de la Recherche Scientifique, Centre Armand-Frappier Santé Biotechnologie, 531 boulevard des Prairies, Laval, Québec, H7V 1B7, Canada; Écosystèmes, Biodiversité, Évolution (ECOBIO), Unité mixte de recherche (UMR) 6553, Centre national de la recherche scientifique (CNRS) - Université de Rennes, Campus Beaulieu, 263 Avenue du Général Leclerc, Rennes, 35042, France; Écosystèmes, Biodiversité, Évolution (ECOBIO), Unité mixte de recherche (UMR) 6553, Centre national de la recherche scientifique (CNRS) - Université de Rennes, Campus Beaulieu, 263 Avenue du Général Leclerc, Rennes, 35042, France; Écosystèmes, Biodiversité, Évolution (ECOBIO), Unité mixte de recherche (UMR) 6553, Centre national de la recherche scientifique (CNRS) - Université de Rennes, Campus Beaulieu, 263 Avenue du Général Leclerc, Rennes, 35042, France; Institut National de la Recherche Scientifique, Centre Armand-Frappier Santé Biotechnologie, 531 boulevard des Prairies, Laval, Québec, H7V 1B7, Canada; Laboratoire de recherche en sciences végétales (LRSV), UMR 5546, Université Paul-Sabatier - CNRS -Institut national polytechnique, 24 chemin de Borde Rouge, Auzeville-Tolosane, 31320, France; Institut National de la Recherche Scientifique, Centre Armand-Frappier Santé Biotechnologie, 531 boulevard des Prairies, Laval, Québec, H7V 1B7, Canada; Écosystèmes, Biodiversité, Évolution (ECOBIO), Unité mixte de recherche (UMR) 6553, Centre national de la recherche scientifique (CNRS) - Université de Rennes, Campus Beaulieu, 263 Avenue du Général Leclerc, Rennes, 35042, France

**Keywords:** plant miRNAs, rhizosphere, bacterial communities, Variovorax, transcriptomics

## Abstract

Small ribonucleic acids (RNAs) have been shown to play important roles in cross-kingdom communication, notably in plant–pathogen relationships. Plant micro RNAs (miRNAs)—one class of small RNAs—were even shown to regulate gene expression in the gut microbiota. Plant miRNAs could also affect the rhizosphere microbiota. Here we looked for plant miRNAs in the rhizosphere of model plants, and if these miRNAs could affect the rhizosphere microbiota. We first show that plant miRNAs were present in the rhizosphere of *Arabidopsis thaliana* and *Brachypodium distachyon*. These plant miRNAs were also found in or on bacteria extracted from the rhizosphere. We then looked at the effect these plants miRNAs could have on two typical rhizosphere bacteria, *Variovorax paradoxus* and *Bacillus mycoides*. The two bacteria took up a fluorescent synthetic miRNA but only *V. paradoxus* shifted its transcriptome when confronted to a mixture of six plant miRNAs. *V. paradoxus* also changed its transcriptome when it was grown in the rhizosphere of *Arabidopsis* that overexpressed a miRNA in its roots. As there were differences in the response of the two isolates used, we looked for shifts in the larger microbial community. We observed shifts in the rhizosphere bacterial communities of *Arabidopsis* mutants that were impaired in their small RNA pathways, or overexpressed specific miRNAs. We also found differences in the growth and community composition of a simplified soil microbial community when exposed in vitro to a mixture of plant miRNAs. Our results support the addition of miRNAs to the plant tools shaping rhizosphere microbial assembly.

## Introduction

Small ribonucleic acids (sRNAs) are thought to play a major role in plant–microbe interactions [1–3]. For example, cotton plants used sRNAs to inhibit the fungal pathogen *Verticillium dahliae* [4] and, similarly, *Arabidopsis thaliana* and tomato used miRNAs to inhibit another fungal pathogen, *Botrytis cinerea* [5–7]. *Arabidopsis* also delivered sRNAs into the oomycete pathogen *Phytophthora capsici* to silence its genes [8]. Similarly, wheat used sRNAs to silence the alpha/beta hydrolase gene in *Fusarium graminearum* [9]. Applying sRNAs targeting *Botrytis* genes on the surface of plants led to an inhibition of the disease [10]. Conversely, microorganisms also use sRNAs to modulate plant gene expression. *B. cinerea* delivered sRNAs to plant cells, silencing the host immune response [11, 12]. Rhizobium delivered transfer RNA-derived sRNAs into soybean cells to regulate nodulation [13]. *Puccinia striiformis* used sRNAs to increase their pathogenicity by suppressing wheat pathogenesis-related genes [14]. Arbuscular mycorrhizal fungi and plants also exchange sRNAs to regulate their mutualistic interactions [15–17]. More recently, rhizosphere microbial sRNAs activated specific pathways in the host plant, playing a role in the soil suppressiveness of *Rhizoctonia solani* [18]. The role of sRNAs in shaping more generally the plant microbiota is, however, not known.

One class of sRNAs that have the potential to shape the microbiota is micro RNAs (miRNAs). miRNAs are small ~21 nt non-coding RNAs that control target gene expression, through sequence complementarity. Their roles in eukaryotes vary from regulating developmental processes to responding to abiotic and biotic stresses. In the mammalian gut, host miRNAs regulated bacterial gene expression and growth, shaping the gut bacterial community [19]. The oral administration of a single miRNA shifted the gut microbiota, mainly by increasing the abundance of *Akkermansia muciniphila* [20]. miRNAs from edible plants, conveyed in exosome-like nanoparticles, were preferentially taken up by gut bacteria and regulated bacterial gene expression [21]. The plant miRNA miR159 was taken up by various gut bacteria and it influenced their gene expression and growth, which led to shifts in the gut microbiota of mice that were fed with this miRNA [22]. In view of these two last studies, it is therefore likely that plant miRNAs also affect the plant microbiota.

**Figure 1:**
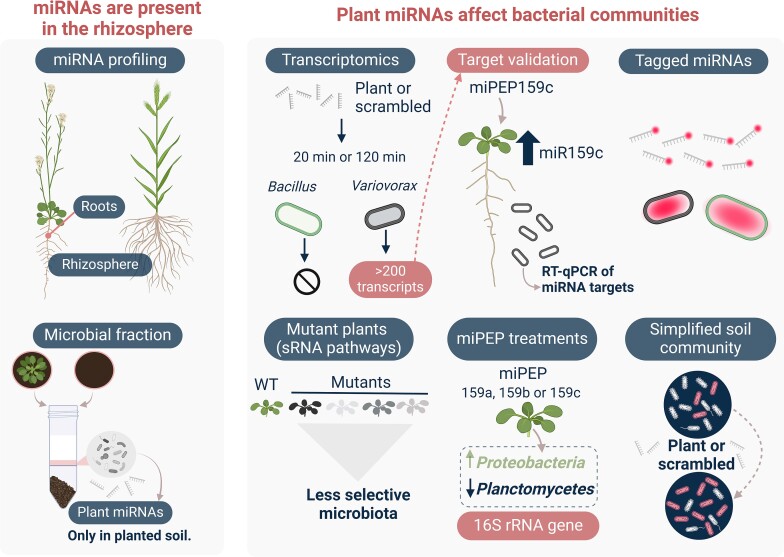
Experimental approach overview. Overview of the experiments leading to the identification of plant miRNAs in the rhizosphere and the confirmation of their effect on microbial communities. Created with BioRender.com

*Arabidopsis* roots contain many miRNAs, of which over half are expressed in a tissue-specific manner, with several being enriched at the root tip, in the early meristematic zone [23]. This zone is also a recognized hotspot for plant-driven microbial selection [24]. The current paradigm is that the plant selects the rhizospheric microbiota through active or passive rhizodeposition, including exudates such as sugars, peptides, amino acids, nucleic acids, nucleotides, fatty acids, or secondary metabolites [25]. We had previously hypothesized that miRNAs could play a role in shaping the rhizosphere microbiota [3]. Here, we hypothesize that plant miRNAs can be found in the rhizosphere of plants and that they influence the gene expression of rhizosphere bacteria, thereby shaping the bacterial community. We report several independent experiments designed to test these hypotheses (Fig. 1).

## Materials and methods

### The full method description is available as supplementary material

#### Detection of plant micro ribonucleic acids in the rhizosphere, roots, and rhizosphere bacteria

For rhizosphere analyses, triplicate *A. thaliana* (Col-0) and *Brachypodium distachyon* (Bd21-3) were grown for a month, alongside unplanted soils. For root analyses, we grew 5 replicated *A. thaliana* (Col-0) plants for 21 days, with unplanted controls. RNA was extracted from the plant roots and rhizosphere and from the unplanted soils. miRNA library preparation with size selection for miRNAs was performed prior to small RNA sequencing. Sequences between 18 and 27 nucleotides were mapped against *Arabidopsis* and *Brachypodium* genomes and were assigned to known miRNAs. This approach ensured that the profiled miRNAs were derived from our model plants. From these miRNAs we constructed an abundance table that was used to compare the rhizosphere and roots to the unplanted controls. Rhizospheric and root miRNAs were defined as the miRNAs that had at least 10 reads for each of the rhizosphere or root samples and a maximum of one read across all the bulk soil samples. Singleton reads were considered to be a sequencing artifact.

We further wanted to show that the plant miRNA had the potential to interact with the rhizosphere bacteria, by showing that they were either on the surface or inside the bacterial cells. We extracted bacterial cells from the rhizosphere soil of 1-month old *A. thaliana* (Col-0) using a Nycodenz gradient. RNA was extracted from the bacterial pellet [26], sequenced, and processed as described above, but with a lower threshold because of the lower number of plant miRNA sequences retrieved. Only the miRNAs that were represented by at least five reads in each rhizosphere sample and absent in the bulk soil samples were kept. To further confirm that miRNAs found in the rhizospheric bacteria originated from the plant and not the bacteria, miRNA sequences were searched for on the + and − strands of 3837 bacterial genomes [27], of which 1160 were isolated from plants.

#### Effect of micro ribonucleic acids on bacterial gene expression

##### Bacterial transcriptomic response to micro ribonucleic acids

*Variovorax paradoxus* EPS and *Bacillus mycoides* YL123 were grown until the exponential phase, when they were exposed to a mix of the six most abundant rhizospheric miRNAs: miR159a, miR159b, miR159c, miR161.1, miR158a, and miR165b, or a control mix of six scrambled miRNAs (same nucleotide composition but in a random order). The cultures were sampled after 20 min and 120 min of incubation, pelleted, and the RNA of the cell pellet was extracted and sequenced. The transcripts were mapped on their respective genomes, and differential expression analyses (plant miRNA vs. scrambled miRNA) were done using DESeq2.

##### *In silico* analysis of micro ribonucleic acids targets

To predict potential targets of the six miRNA used on the genome of *V. paradoxus*, we implemented a workflow [28] named “mirnatarget 1.0: miRNA target finder” (https://github.com/jtremblay/MiRNATarget), which was inspired from the plant miRNA target finder, “psRNAtarget” [29].

##### Confocal microscopy

We then wanted to know if the difference between the response of *V. paradoxus* EPS and *B. mycoides* YL123 was due to a barrier blocking entry into the cell. We grew the two bacteria overnight in liquid media. The cultures were incubated for 4 h with 3’-Cy5 fluorescent miRNAs (ath-miR159a or a scrambled control—same nucleic acid content but in different order), or a pCp-Cy5 control at a final concentration of 2 μM. Twenty minutes before visualization, the cultures were also treated with MitoTracker Green FM (Invitrogen), which stains all live bacteria. We visualized the washed and concentrated culture using a confocal microscope (Zeiss LSM780).

##### Flow cytometry

We prepared the cultures as described in the confocal microscopy section. The cells were fixed with 4% paraformaldehyde and then stained with a deoxyribonucleic acid (DNA) marker (Hoechst 33342). The cells were diluted in PBS and processed by flow cytometry (BD LSRFortessa). We used a variety of controls to ensure that our statistical analyses were conducted on bacteria that were positive for our green (MitoTraker), red (Cy5 tagged miRNAs), and blue (Hoechst) markers.

##### *In* vitro micro ribonucleic acid-encoded peptide treatment and transcriptomic experiment

To confirm *in planta* the transcriptomic results for *V. paradoxus*, ~50 *A. thaliana* (Col-0) surface-sterilized seeds were grown axenically in Petri dishes in a growth chamber. After 20 days of growth, the plants were treated twice within 24 h by inoculating miPEP159c, a scrambled micro ribonucleic acid-encoded peptide (miPEP), or water at the plant crown. One hour after the second miPEP treatment, *V. paradoxus* was inoculated along the roots of the seedlings. Two hours after the bacterial inoculation, the plants and rhizospheres were sampled, and RNA was extracted. From the potential targets of miR159c found above, three were selected for reverse transcriptase real-time quantitative PCR (RT-qPCR) analyses in the rhizosphere—alpha-2-macroglubulin, phosphatidate cytidylyltransferase (CdsA), and LysR. We also quantified the abundance in plant tissues of the primary transcript of mir159c, pri-miR159c.

#### Effect of micro ribonucleic acids on the bacterial community

##### *Arabidopsis* mutant experiment

Five *A. thaliana* mutants were chosen. *RTL1* mutant overexpresses RTL1 protein which results in a suppression of siRNA pathway without affecting miRNAs [30]. *RTL1myc* overexpresses RTL1 protein flagged with Myc epitope, rendering RTL1 less active, so siRNA pathway is less suppressed than with *RTL1* mutant. *Ago1-27* mutant has AGO protein function partially impaired and is completely post-transcription gene silencing (PTGS) deficient [31]. *Dcl1-2* mutant has total loss of function of DCL1 protein resulting in low levels of miRNA and developmental problems [32]. *Hen1-4* mutant is miRNA defective but is also affected in some siRNA–PTGS [33]. HEN1 methylates siRNA and miRNA not only to maintain their levels and size but also to protect them from uridylation and subsequent degradation [34]. The plants were grown for a month, after which the roots and attached rhizosphere were sampled, their DNA extracted, amplified using 16S ribosomal RNA (rRNA) gene primers, and sequenced. The same primers were used in real-time quantitative PCR to quantify bacterial abundance. Amplicon sequencing data was processed with AmpliconTagger [35] and the R package “phyloseq” v 1.32.0 [36].

##### Micro ribonucleic acid-encoded peptide experiment

We treated *A. thaliana* Col-0 with 500 μL of water (control condition) or a miPEP solution (20 μM of miPEP159a, miPEP159b, or miPEP159c), applied at the base of the crown, 3 times a week for a total of 10 applications. We then extracted the DNA from the roots attached rhizosphere and sequenced and quantified the 16S rRNA gene as described above.

##### Simplified soil community laboratory experiment

We created, *in vitro*, a simplified soil community by inoculating five different growth media with 2 g of agricultural soil. The cultures were normalized to the same optical density, pooled, pelleted, and suspended in PBS. The cells were inoculated in a 96-wells plate containing a mixture of 17 amino acids as nitrogen source. Five wells were treated with a mixture of rhizospheric miRNAs (ath-miR158a-3p, ath-miR158b, ath-miR159a, ath-miR827, and ath-miR5642b) and five wells were treated with a mixture of scrambled miRNAs. These miRNAs were all found in the rhizosphere (but for some below the stringent threshold used above) and were predicted to target bacterial genes associated with nitrogen cycling. We measured bacterial growth every hour for 52 h, after which we sampled the bacteria, extracted the DNA, amplified and sequenced the 16S rRNA gene as described above.

## Results

### Plant micro ribonucleic acids are present in the rhizosphere

**Figure 2:**
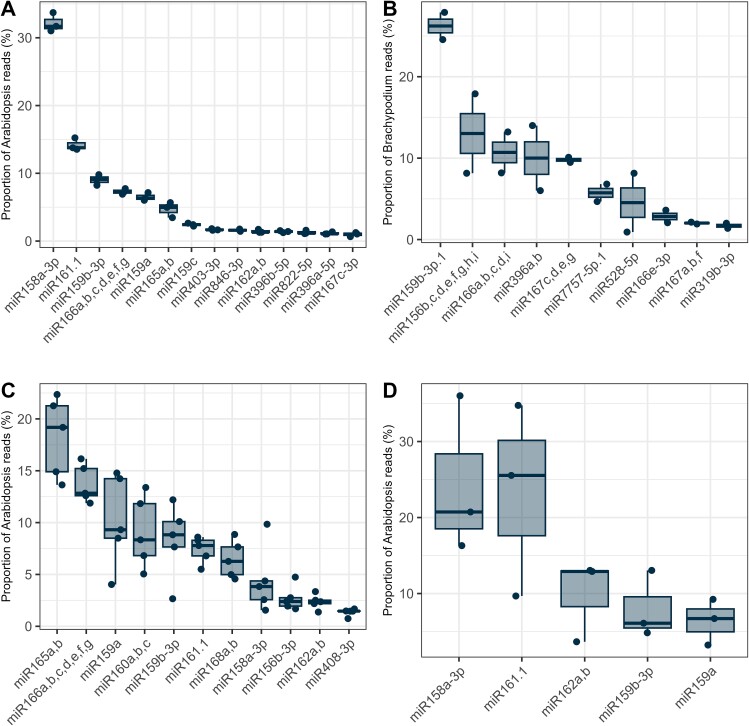
Plant miRNAs are present in the rhizosphere, roots, and bacteria and absent in unplanted soil. Relative abundance of the plant miRNAs found in the rhizosphere of (A) *A. thaliana*, (B) *B. distachyon*, (C) in the roots of *A. thaliana*, and (D) in or on rhizosphere bacteria while completely absent from unplanted soil.

We sequenced small RNA extracted from *A. thaliana* rhizosphere soil and unplanted soils. One-hundred-eleven ath-miRNAs (mapped on *A. thaliana*’s genome) were detected, of which 14 were present in the rhizosphere with >10 reads per sample and absent in unplanted soil (Fig. 2A). The most abundant miRNAs in the rhizosphere were ath-miR158a-3p, ath-miR161.1, and various members of the miR159, miR166, and miR165 families (Fig. 2A). We then sequenced the rhizosphere miRNAs of a second model plant, *B. distachyon*. Out of the 81 bdi-miRNAs (mapped on *B. distachyon*’s genome) detected in the rhizosphere, 10 were represented by >10 reads per sample and absent in unplanted soil (Fig. 2B). The most abundant miRNAs were bdi-miR159b-3p, bdi-miR156, bdi-miR166, bdi-miR396, and bdi-miR167. Among the rhizospheric miRNAs detected above our threshold, four were common between *A. thaliana* and *B. distachyon*: miR159b-3p, miR167, miR166, and miR396.

We then grew more *Arabidopsis* plants and sequenced their root miRNAs to confirm that the rhizospheric miRNAs could be coming from the plant roots. Eleven miRNAs were represented by at least 10 reads in each root sample. There was a clear dominance of ath-miR165, ath-miR166, ath-miR159a, ath-miR160, and ath-miR159b.3p (Fig. 2C). Among these 11 miRNAs, 7 were common with the miRNAs found in *Arabidopsis* rhizosphere (ath-miR158a-3p, ath-miR159a, ath-miR159b-3p, ath-miR161.1, ath-miR162, ath-miR165, ath-miR166), which included most of the top 5 most abundant root and rhizosphere miRNAs.

To confirm that plant miRNA could interact with rhizosphere bacteria, bacterial cells were isolated from the rhizosphere of 1-month-old *A. thaliana*, washed and their RNA content was extracted and sequenced. The miRNAs were mapped against *A. thaliana* TAIR10.1 genome, identifying a total of 34 ath-miRNAs. Five miRNAs—namely ath-miR158a-3p, ath-miR161.1, miR162, ath-miR159b-3p, and ath-miR159a—were represented by at least five reads per rhizosphere samples and absent in bacteria extracted from the unplanted soil (Fig. 2D). Four out of these five miRNAs were among the five most abundant miRNAs found in the rhizosphere of *A. thaliana* (Fig. 2A) and were found in a similar rank-abundance order. To ensure that these miRNAs did not come from the bacteria themselves, their gene-encoding sequences were searched for in 3837 soil bacterial genomes, of which 1160 were of bacteria isolated from plants [27]. No matches were found, meaning that the miRNAs detected in the bacteria could not be produced by the bacteria.

### Plant micro ribonucleic acids shift rhizosphere bacterial gene expression

**Figure 3:**
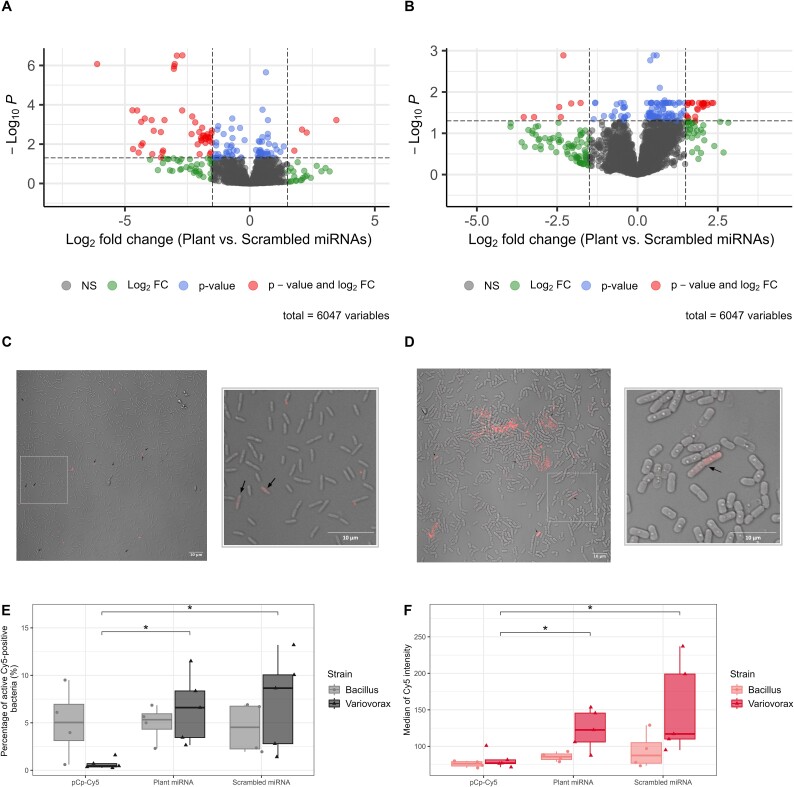
Plant miRNAs affect the transcriptome of a rhizosphere bacterium. Gene expression of *Variovorax paradoxus* after (A) 20 min and (B) 120 min exposure to a mixture of six synthetic miRNAs (plant miRNAs compared to scrambled miRNAs), confocal microscopy images of (C) *V. paradoxus* and (D) *B. mycoides* after a 4-h exposure to ath-miR159a tagged with Cy5 and flow cytometry experiment showing (E) the percentage of active bacterial cells positive for Cy5 signal and (F) the median intensity of the Cy5 signal in active bacterial cells. Arrows in (C) and (D) indicate cells containing the fluorescent molecule. *: *P* < .05.

We incubated *V. paradoxus* EPS [37] and *B. mycoides* [38] with a synthetic mixture of the six most abundant *A. thaliana* rhizosphere miRNAs: miR159a, miR159b, miR159c, miR161.1, miR158a and miR165b, or a mixture of scrambled miRNAs (same nucleotide composition but in a random order) at the same concentration. *Bacillus* did not respond to the treatment—no gene was significantly differentially expressed following incubation with the synthetic miRNAs. In contrast, *Variovorax* showed important changes in response to the miRNA confrontation, as revealed by a differential abundance analysis of plant miRNA vs. scrambled miRNA treatments. After 20 min of incubation, the expression of 79 genes was significantly lower in the plant miRNA-treated cultures and the expression of 44 genes was significantly higher (adjusted *P* < .05, Fig. 3A and Supplementary Table S1). After 120 min of incubation, the expression of 24 genes was significantly lower in the plant miRNA-treated cultures and the expression of 104 genes was significantly higher (adjusted *P* < .05, Fig. 3B and Supplementary Table S1). Many genes were repressed after 20 min following the addition of the synthetic plant miRNAs to the bacterial culture, whereas after 120 min, more genes presented an increased expression. Only one gene was differentially expressed at both time points, a gene coding for a methionine synthase (VARPA_RS01000), which was overexpressed in response to the plant miRNA treatment.

Using the rules of a plant small RNA target finder, psRNAtarget, we compared the differentially expressed genes from the previous experiment with the predicted target genes for the six miRNAs used. The six miRNAs were predicted to target 237 sequences in the *V. paradoxus* EPS genome. Among these, 100 targets were positioned too far from any coding sequence (CDS), so they were removed from the following analysis, resulting in 137 potential targets. Among the 123 genes differentially expressed at 20 min, only 2 were predicted as targets *in silico*: VARPA_RS05960 (targeted by miR165), coding for an L-iditol 2-dehydrogenase, and VARPA_RS26385 (targeted by miR159a, b and c), coding for a CDP-diacylglycerol synthase (CdsA). Only two genes were predicted as targets among the 128 differentially expressed genes at 120 min: VARPA_RS00680 (targeted by miR165), coding for a hypothetical protein, and VARPA_RS22555 (targeted by miR158a-3p and miR159c), coding for a non-ribosomal peptide synthetase.

To test if the differences in sensitivity between *V. paradoxus* and *B. mycoides* were due to a barrier blocking the entry into the cell, we exposed them to a Cy5-tagged synthetic ath-miR159a and visualized its localization using confocal microscopy. Images show a clear localization of the miRNA inside many bacterial cells (Fig. 3C and D). Flow cytometry confirmed that an average of 6.51% *Variovorax* cells contained the Cy5 signal from plant miRNAs (average median fluorescence intensity = 123), compared to 4.95% of *Bacillus* cells (average median fluorescence intensity = 85.75; Fig. 3E and F). The scrambled miRNA, containing the same nucleotides as ath-miR159a but in a different order, was internalized as efficiently (Supplementary Fig. S1 and Fig. 3E), suggesting a general sequence-independent internalization mechanism for miRNAs. *Variovorax*, in contrast to *Bacillus*, internalized more efficiently the tagged plant miRNAs than the tagged single nucleotide. Indeed, a comparable amount of pCp-Cy5 was also internalized by *Bacillus* (on average 5.04%, average median fluorescence intensity = 75.8), but this was an order of magnitude lower for *Variovorax* (on average 0.67%, adjusted *P* = .0200, average median fluorescence intensity = 81.5, adjusted *P* = .0355; Fig. 3E and F and Supplementary Fig. S1).

**Figure 4:**
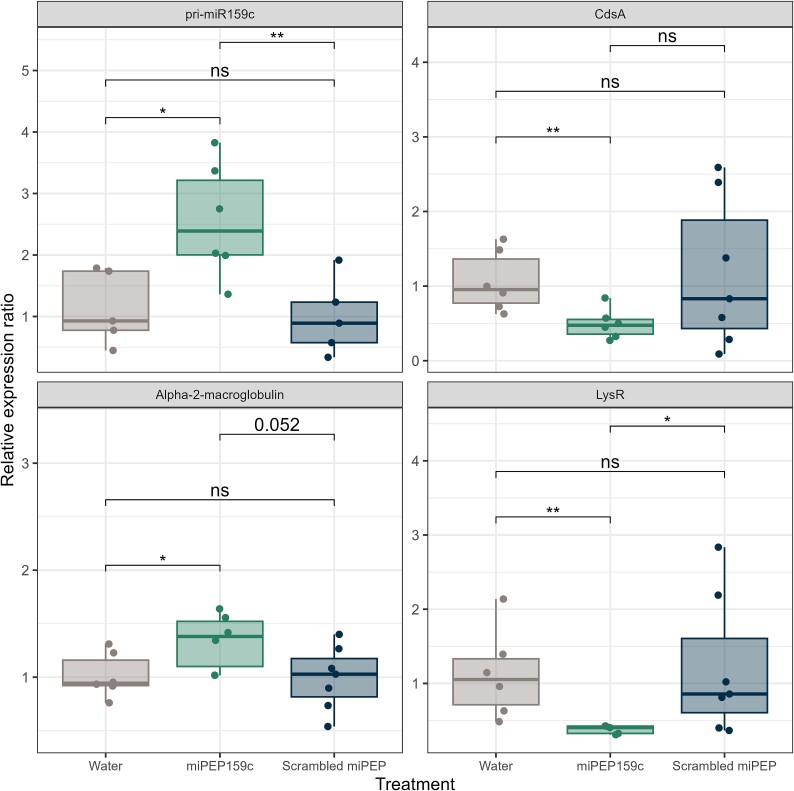
miPEP 159c shifts the expression of *V. paradoxus*. Expression of pri-miR159c in A. thaliana roots and *V. paradoxus* expression of CdsA, alpha-2-macroglobulin and LysR in the rhizosphere after exposure of the plant to miPEP159c vs. a water and a scrambled miPEP controls. ns: *P* > .05; *: *P* < .05, **: *P* < .01.

To confirm the effect of miRNAs on the bacterial transcriptome *in planta*, we exposed Arabidopsis plants growing in vitro to the miRNA-encoded peptide (miPEP) miPEP159c and then inoculated them with *Variovorax*. miPEPs increased the expression of specific plant miRNAs [39, 40]. We selected miR159c because it was among the most abundant miRNAs in the rhizosphere, was in the mixture of miRNAs that modulated the gene expression of *Variovorax*, and was predicted to target several key genes. The relative expression of the corresponding primary transcript (pri-miR159c) in the *Arabidopsis* plant tissue increased by a factor 1.58 as compared to the scrambled miPEP control (same amino acid composition as the miPEP, but in different order; *t*-test: t = 3.33, *P* = .00929; Fig. 4). We then quantified the expression of three *Variovorax* genes determined to be potential targets of the miR159c according to our bioinformatic and transcriptomic analyses. One hundred and twenty minutes after the miPEP159c application, the relative expression of the LysR genes decreased by a factor 0.69 (*t*-test: t = −3.06, *P* = .0195, Fig. 4), whereas the expression of alpha-2 macroglobulin gene increased by a factor 0.34 (*t*-test: t = 2.18, *P* = .0520, Fig. 4) in comparison with the scrambled miPEP control. The expression of the CdsA gene did not differ between the miPEP159c and the scrambled miPEP control (Fig. 4). The relative expressions of the three genes and the pri-miR159c following the application of the miPEP159c were all significantly different from the water control, whereas it was never the case for the scrambled miPEP (Fig. 4).

### Plant micro ribonucleic acids influence the rhizosphere bacterial community

**Figure 5:**
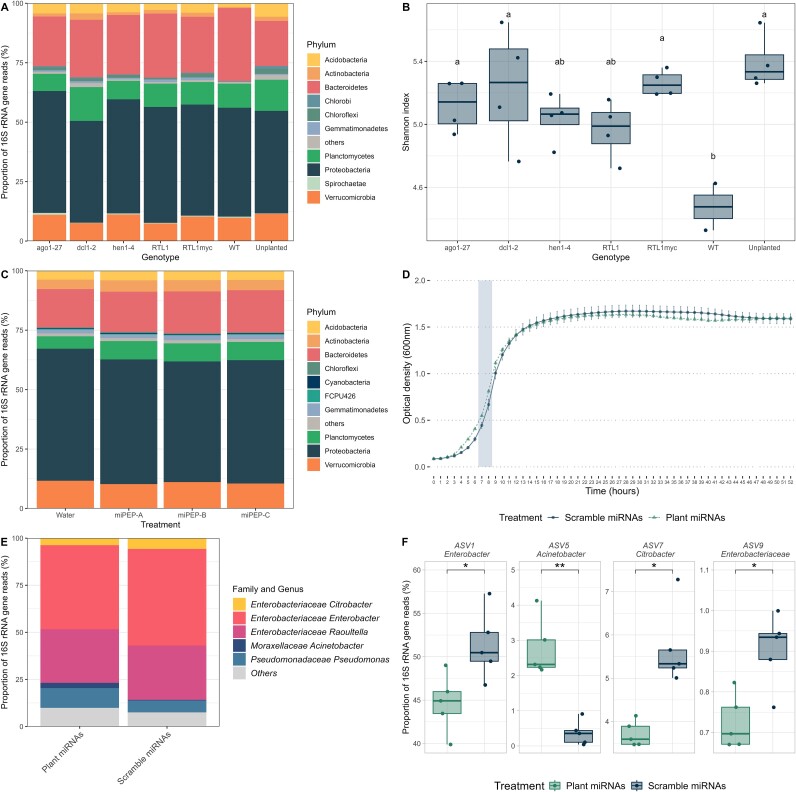
Plant miRNAs affect the bacterial community. (A) Phylum-level bacterial community composition and (B) Shannon diversity for different *Arabidopsis* mutants with impaired small RNAs pathways. (C) Phylum-level bacterial community composition for *Arabidopsis* plants inoculated with miPEP159a, miPEP159b, or miPEP159c. (D) Growth curve, (E) community composition at genus level, and (F) relative abundance of significantly affected ASVs for a simplified soil microbial community exposed to a mixture of synthetic plant or scrambled miRNAs. *: *P* < .05, **: *P* < .01. Different letters in (B) indicate significant differences (P<.05) in Tukey HSD tests.

To investigate the role of plant small RNAs on the rhizospheric microbial diversity, we grew *A. thaliana* mutants with disturbed miRNA and/or siRNA biosynthesis pathways and analyzed their rhizospheric microbial communities by 16S rRNA gene amplicons sequencing. The rhizosphere bacterial communities varied across the different genotypes (permutational multivariate analysis of variance [PERMANOVA]: *P* < .05; Fig. 4A). In principal coordinates analysis (PCoA) ordinations, *ago1-27* and *RTL1myc* mutants’ communities were more like unplanted soil communities than those of wild-type (WT) plants (not shown). In subsequent pairwise PERMANOVA, there were significant differences between the community composition of the following mutant pairs: *ago1-27* and *hen1-4*, *ago1-27* and *RTL1*, *hen1-4* and *RTL1myc*, and *RTL1* and *RTL1myc*. The microbial community of WT plants was nearly significantly different (*P* < .10) from all the mutants. These differences were mirrored in the community composition at the phylum level, with significant differences between the genotypes for the relative abundance of *Acidobacteria* (*F* = 7.8, *P* = .00856), *Actinobacteria* (*F* = 3.2, *P* = .0363), *Chloroflexi* (*F* = 5.7, *P* = .00391), *Planctomycetes* (*F* = 5.3, *P* = .00534), *Spirochaete* (*F* = 3.6, *P* = .0255), and *Verrucomicrobia* (*F* = 6.4, *P* = .00231; Fig. 5A). For the *Acidobacteria*, *Actinobacteria*, and *Chloroflexi*, most of these differences were due to differences between the WT plants and some or all the mutants (Fig. 5A), according to post-hoc Tukey honestly significant difference (HSD) tests. For the other phyla, the differences were rather between different mutants (Fig. 5A). Bacterial diversity was higher in the rhizosphere of most mutant plants and in the unplanted soil as compared with the rhizosphere of the WT plants, whereas there was no difference between the rhizosphere of the mutants and the unplanted controls (Fig. 5B).

We then treated soil-grown *Arabidopsis* with miPEPs to further test for the effect of the overexpression of specific miRNAs on the rhizospheric bacterial community. The application of three miPEPs (miPEP159a, miPEP159b, and miPEP159c) on the crown of *Arabidopsis* growing in soil changed the bacterial community in the roots/rhizoplane (PERMANOVA: *R*^2^ = 0.160, *P* = .031). All pairwise comparisons with the water control were significant or nearly significant (pairwise PERMANOVA: miPEP159a *R*^2^ = 0.11, adjusted *P* = .083; miPEP159b *R*^2^ = 0.16, adjusted *P* = .025; miPEP159c *R*^2^ = 0.15, adjusted *P* = .033). At the phylum level, the application of the miPEPs increased the relative abundance of the *Proteobacteria* (*F* = 8.9, *P* = .00029) and decreased the relative abundance of the *Planctomycetes* (*F* = 40.8, *P* = 3.64 × 10^−10^; Fig. 5C). This was due to significant (adjusted *P* < .05) differences between the water control and each of the miPEP treatments in post-hoc Tukey HSD tests. The application of the miPEP on the crown of *Arabidopsis* did not, however, affect the bacterial alpha diversity nor abundance.

Finally, we subjected in vitro a simplified microbial community, that was enriched from an agricultural soil, to a mix of five synthetic plant miRNAs (ath-miR158a-3p, ath-miR158b, ath-miR159a, ath-miR827, and ath-miR5642b), or a mix of their scrambled counterparts. Exposure to the plant miRNAs significantly disturbed the growth of the microbial communities during the log phase (*t*-test: *P* < .05, Fig. 5D). The bacterial community contained 20 amplicon sequence variants (ASVs) at the endpoint of the incubation (52 h) and we found significant shifts in the bacterial community composition due to the plant miRNA exposure (PERMANOVA: *R*^2^ = 0.39, *P* = .003, Fig. 5E). At the genus level, the plant miRNAs increased the relative abundance of the *Acinetobacter* and decreased the relative abundance of the *Citrobacter* and *Enterobacter* (Fig. 5E). At the ASV level, plant miRNAs decreased the abundance of three ASVs related to the genera *Enterobacter* (*P* = .0208) and *Citrobacter* (*P* = .0184) and to the *Enterobacteriaceae* family (*P* = .0184) and increased the relative abundance of an ASV related to the genus *Acinetobacter* (*P* = .00604; Fig. 5F).

## Discussion

Using multiple lines of independent evidence, we confirmed our hypotheses. Plant miRNAs are present in the rhizosphere, and they induce changes in the transcriptome of a rhizosphere bacterium, leading to shifts in the bacterial community. Plants and microorganisms are known to interact using small RNAs [4–14, 18] and the gut microbiota was shown to be shaped by miRNAs, including plant miRNAs [19–22], but this is the first report of this mechanism for plant-bacterial community interactions in the rhizosphere.

The two model plants, *A. thaliana* and *B. distachyon* harbored a similar complement of plant miRNAs in their rhizospheres. Although it would require confirmation from more plant species, the presence of similar miRNAs in the rhizosphere of a dicotyledon and a monocotyledon suggests a conserved feature among land plants. All the major miRNAs that we found in the rhizosphere of *Arabidopsis* were also detected in the roots. This agrees well with previous reports of root miRNAs, where the two most abundant rhizospheric miRNAs found in our study—ath-miR158a and ath-miR161.1—were reported to be highly enriched in the early meristematic zone of the roots [23]. Many root exudates, such as extracellular DNA, soluble compounds, and mucilage, are produced and secreted, by border cells, in this region of the root tip [41]. Even though there was a large overlap between the root and rhizosphere miRNAs in our two experiments, the rank abundance of the miRNAs was not the same, alluding to a potential selection mechanism for the miRNAs that make it to the rhizosphere. Alternatively, this pattern could also be explained by different half-lives in the rhizosphere, preferential uptake by bacteria, the use of entire roots for sequencing, or by the slightly different conditions under which the two experiments were run. Finally, bacteria growing in the rhizosphere of *Arabidopsis* harbored plant miRNA inside or outside their cells, confirming that the rhizosphere miRNAs reached the rhizosphere bacteria.

*In planta*, in the same way that some bacteria secrete small RNAs in outer membrane vesicles [42], bacteria may internalize external DNA *via* vesiduction [43], i.e. membrane fusion of a vesicle containing DNA or RNA. The use of vesicles seems, however, not necessary for plant-microbe miRNA-based interactions, as exposure to naked miRNAs led to transcriptomic and community shifts. The uptake of naked or vesicle-borne miRNAs was already shown for the gut microbiota [19–22] and is now generally accepted. The incorporation of eukaryote miRNAs in bacteria is also consistent with their ability to absorb environmental nucleic acids, such as extracellular DNA, through natural competence [44]. It is, however, difficult to measure the total concentration of miRNA in the rhizosphere, and how that compared to the amounts used for our in vitro experiments. *In vitro*, we used concentrations previously used for gut microbiota studies [19], but we are not sure if this corresponds to the concentrations usually found in the rhizosphere.

In plants, miRNA induce mRNA cleavage or translation inhibition, through near perfect sequence complementarity [45]. Studies in the human gut also suggested that host miRNAs interact with bacterial mRNA through sequence complementarity [19, 21], so we used the rules for plant miRNA based on sequence homology, to search for targets in bacterial genomes. We also used this model to select the genes targeted by ath-miR159c in the *V. paradoxus* genome, which expression was quantified by RT-qPCR in *Variovorax* growing in the rhizosphere of gnotobiotic *Arabidopsis* treated with miPEP 159c. We found that one out of three targets was indeed inhibited following miPEP 159c application and increased expression of the primary transcript of miR159c. The third gene, encoding for alpha-2-macroglobulin, was, however, overexpressed in the presence of the miPEP. In bacteria, non-coding small RNAs can sometimes induce expression of target mRNAs [19, 42]. Even though this gnotobiotic experiment showed that bioinformatic prediction tools worked relatively well when focusing on a few genes, *V. paradoxus* differentially expressed only 4 of the 137 predicted targets and differentially expressed another 247 non-predicted genes when exposed in liquid cultures to a mixture of six rhizospheric miRNAs as compared to a control mixture of six corresponding scrambled miRNAs. Such a low level of overlap is comparable to what we would expect from two random set of genes. This suggests that either (i) the bacteria adjusted their transcriptome in response to the shift in the expression of the miRNA-targeted gene, (ii) as it is often the case in plants [46], the miRNAs targeted bacterial transcription factors, such as the ones from the LysR family that were differentially expressed at both time points, (iii) target genes were translationally repressed, which would be undetectable with transcriptomics, though this is less common in plants than mRNA cleavage [45], (iv) miRNAs protected targets from repression, as it was shown for arbuscular mycorrhizal fungi [47], or (v) because many predicted targets of the rhizospheric miRNAs were not in CDS, miRNAs could have affected DNA methylation [48] or interacted with gene promoters [49]. Clearly, the bioinformatic tools used were not appropriate to predict the full complement of genes that were affected by the miRNAs. Several supplementary experiments will be needed to confirm the targets and mechanism of action of plant miRNA in bacteria. Among other approaches [50], reporter gene assays [22, 51], biochemical fishing [52, 53], in vivo RNA proximity ligation [54], 5′-RLM RACE [55] and mutant bacteria with resistant targets could be envisioned in further studies.

In contrast to *Variovorax*, plant miRNAs did not impact the transcriptome of *Bacillus*. In our simplified soil community, only 4 out of the 20 ASVs were significantly impacted by plant miRNAs, and, similarly, intestinal miRNAs only impacted the growth of specific bacterial strains [19]. Plant exosomes containing miRNAs are also preferentially taken up by some bacteria, affecting their gene expression and activity [21]. Our experiments were, however, carried out using naked miRNAs, excluding this explanation. After the transcriptomic experiment, our hypothesis was that the different responses between the two bacteria could be related to differences in the cell wall that made miRNA entry impossible for Gram-positive. But our microscopy work disproved that. Both our Gram-positive and Gram-negative model bacteria showed that they could take up the miRNAs. Another possibility is that the mechanism of interaction differs between the two groups of bacteria. In bacteria, Argonaute homologs [56, 57] and chaperone proteins such as RNA-binding Hfq, ProQ, or CrsA proteins protect small RNAs and stabilize their interaction with mRNA, improving the formation of sRNA-mRNA duplexes that lead to gene silencing [58]. This crucial role of chaperones in sRNA-mediated interactions was mostly reported for Gram-negative bacteria and only for a handful of Gram-positive bacteria [59, 60]. Alternatively, competence for DNA uptake depends on environmental conditions, such as stress, nutrient availability, and cell density [44]. The two bacteria tested might have different cues to initiate nucleic acid uptake, and the growing conditions might not have been met during the transcriptomic experiment to trigger this behavior in *Bacillus*. Another possibility is that these bacteria employ distinct sRNA turnover mechanisms through the action of RNA nucleases such as, RNase III, RNase E, and PNPase, which accelerate sRNA turnover [61, 62]. In any case, the differential transcriptomic response to miRNAs of the two bacteria tested suggests a selective mechanism in the rhizosphere. Any effect on a bacterium could have cascading effects on the rest of the community.

*Arabidopsis* miRNAs impacted the bacterial community in the root environment. First, we examined the root-associated bacterial community of *A. thaliana* mutants affected in the biosynthesis of miRNA and/or sRNA. Many of these mutants had disrupted bacterial communities compared to WT plants. One of the most relevant mutants, the *dcl1-2* mutant, which is specifically impaired in miRNA production, was severely affected in its community composition at the phylum level and harbored a more diversified community than the WT plants. The microbial community composition and diversity in the roots and rhizosphere of the mutants resembled those of an unplanted soil more than those of WT plants. This suggests that mutations in small RNA related pathways lead to a weaker selective pressure in the roots and rhizosphere of the mutants. The mutations used are, however, pleiotropic, and plants were severely affected in their phenotype, which could have also affected the bacterial community.

Second, the bacterial community associated with soil-grown *Arabidopsis* responded significantly to miPEP application. As we showed in the in vitro experiment using miPEP159c, miPEPs stimulate the production of their corresponding miRNA in plant tissues. This means that the upregulation of a single miRNA could lead to changes in the bacterial community. Other than the direct effect of the overexpressed miRNA on the bacteria, the shifts observed in bacterial community could, however, also be explained by other factors. For instance, plant miRNAs alter various physiological processes within the plant, such as root development and plant immune response [46].

Third, to exclude most of the unavoidable indirect plant-mediated effects of the mutant and the miPEP experiments, we did an in vitro experiment with a simplified soil-derived bacterial community of twenty ASVs exposed to a mixture of synthetic *Arabidopsis* miRNAs. The plant miRNAs affected the abundance of four ASVs and the growth of the community during the log phase, as compared to a mixture of scrambled miRNAs. This shows that plant miRNAs directly affect bacterial communities. At the individual level, a bacterium could change in relative abundance because of (i) direct effect of the miRNA on its growth or (ii) changes in the relative abundance of other bacteria with which it interacts. Some species can have a keystone role in interaction networks [63], and a shift in these species would influence the entire community. For instance, the presence of *Variovorax* in the rhizosphere of *Arabidopsis* counteracted the root growth inhibition induced by many other members of the community [64]. Plant miPEPs, through their effect on miRNAs, also modulated the interactions between plants and key root symbionts, such as arbuscular mycorrhizal fungi [47] and rhizobia [65]. This effect would profoundly alter the microbial community, even if the miRNAs had only affected a single or a few keystone species.

We showed here for the first time that plant miRNAs are found in the rhizosphere of two model plants and that they affect the transcriptome of a rhizosphere bacterium, and that they modulate soil microbial community composition and growth. The rhizosphere effect is thought to be mainly due to the rhizodeposition of various small organic molecules. Our study shows that miRNAs should be added to the molecules that plants use to interact with their rhizosphere microbiota.

## Supplementary Material

figS1_ycae120

SupplementaryTableS1_ycae120

SupplementaryTableS2_ycae120

SupplementaryTableS3_ycae120

20240510SuppMethods_ycae120

## Data Availability

We deposited the sequence data generated in this study under NCBI BioProject accessions PRJNA836586 (rhizosphere miRNAs), PRJNA1107220 (root miRNAs), PRJNA1111839 (soil-extracted bacteria miRNAs), PRJNA1111831 (mutant and miPEP 16S rRNA gene amplicons), PRJNA1111829 (simplified soil community 16S rRNA gene amplicons), and PRJNA1111827 (*Bacillus* and *Variovorax* transcriptomics). The R code used to manipulate the data and generate the figures is available on GitHub (https://github.com/le-labo-yergeau/Middleton_miRNA) with the accompanying data being available on Zenodo (https://zenodo.org/doi/10.5281/zenodo.11105307).
