# Correction to: Rhizospheric miRNAs affect the plant microbiota

**DOI:** 10.1093/ismeco/ycae144

**Published:** 2024-12-04

This is a **correction** to:

Harriet Middleton, Jessica Ann Dozois, Cécile Monard, Virginie Daburon, Emmanuel Clostres, Julien Tremblay, Jean-Philippe Combier, Étienne Yergeau, Abdelhak El Amrani, Rhizospheric miRNAs affect the plant microbiota, *ISME Communications*, Volume 4, Issue 1, January 2024, ycae120, https://doi.org/10.1093/ismeco/ycae120

In the originally published PDF version of this manuscript, the volume number in which the article was published was incorrect. The publisher apologizes for the error, which has been corrected.

