# Supplementary material for: Rhizospheric miRNAs affect the plant microbiota
Source: ISME Commun. 2024 Oct 12;4(1):ycae120. doi: 10.1093/ismeco/ycae120 (PMC11520407; doi:10.1093/ismeco/ycae120)
Supplement: SupplementaryTableS1_ycae120 [file supplementarytables1_ycae120.pdf]

Gene	Mean expression	log2 fold change	log2 fold change SE	stat	p-value	adjusted P-value	kegg_definition
20 minutes							
gene-VARPA_RS00165	3011.028	-0.394	0.087	-4.535	5.75E-06	0.001507097	P-type Cu+ transporter
gene-VARPA_RS00975	1508.973	0.406	0.118	3.436	0.000591382	0.035272106	hypothetical protein
gene-VARPA_RS01000	12389.710	0.387	0.106	3.663	0.000249169	0.020561532	5-methyltetrahydrofolate--homocysteine methyltransferase
gene-VARPA_RS01415	95.845	0.998	0.303	3.297	0.000976924	0.049041587	biuret amidohydrolase
gene-VARPA_RS01850	1664.679	1.355	0.357	3.798	0.000145619	0.01309268	fumarylpyruvate hydrolase
gene-VARPA_RS01860	4479.233	0.900	0.274	3.284	0.001024586	0.04989841	hypothetical protein
gene-VARPA_RS02485	449.016	-1.240	0.375	-3.310	0.000931583	0.048095594	hypothetical protein
gene-VARPA_RS02995	580.180	0.643	0.108	5.955	2.59E-09	2.23E-06	two component transcriptional regulator, winged helix family
gene-VARPA_RS03015	2108.631	1.220	0.340	3.586	0.000335887	0.024675415	glyoxylate/hydroxypyruvate reductase
gene-VARPA_RS04335	4281.141	-1.523	0.343	-4.436	9.17E-06	0.002046866	NADH:quinone reductase (non-electrogenic)
gene-VARPA_RS04355	3379.881	-1.351	0.352	-3.836	0.000125012	0.011604285	fatty-acyl-CoA synthase
gene-VARPA_RS04360	1121.924	-2.307	0.467	-4.940	7.81E-07	0.000391948	hypothetical protein
gene-VARPA_RS04365	1829.633	-2.187	0.465	-4.700	2.60E-06	0.00078356	L-carnitine dehydratase/bile acid-inducible protein F
gene-VARPA_RS04370	1629.035	-1.937	0.479	-4.045	5.23E-05	0.006301188	Enoyl-CoA hydratase/isomerase
gene-VARPA_RS04375	2046.405	-1.935	0.480	-4.028	5.62E-05	0.006637571	acyl-CoA dehydrogenase domain-containing protein
gene-VARPA_RS04380	2854.078	-1.828	0.431	-4.241	2.23E-05	0.003627734	acyl-CoA dehydrogenase domain-containing protein
gene-VARPA_RS04385	3099.633	-1.671	0.404	-4.132	3.59E-05	0.004805128	acetyl-CoA C-acetyltransferase
gene-VARPA_RS04390	3778.601	-1.800	0.433	-4.154	3.27E-05	0.004640389	3-hydroxyacyl-CoA dehydrogenase
gene-VARPA_RS04635	2265.245	0.429	0.117	3.664	0.000248515	0.020561532	hypothetical protein
gene-VARPA_RS04640	702.980	0.509	0.145	3.519	0.000433787	0.029034786	muconolactone D-isomerase
gene-VARPA_RS04645	865.618	0.490	0.123	3.989	6.63E-05	0.007400665	maleamate amidohydrolase
gene-VARPA_RS04775	477.933	0.434	0.122	3.565	0.000363215	0.025741272	protein of unknown function DUF894 DitE
gene-VARPA_RS04800	2897.921	0.376	0.104	3.598	0.000320439	0.024675415	23S rRNA (cytosine1962-C5)-methyltransferase
gene-VARPA_RS04990	21459.393	3.460	0.722	4.795	1.62E-06	0.000600232	3-deoxy-7-phosphoheptulonate synthase
gene-VARPA_RS04995	10461.811	2.273	0.522	4.356	1.33E-05	0.00257733	uncharacterized protein
gene-VARPA_RS05425	42479.627	-6.112	0.986	-6.201	5.62E-10	8.46E-07	indolepyruvate ferredoxin oxidoreductase
gene-VARPA_RS05485	1123.283	0.507	0.098	5.169	2.36E-07	0.000177662	alkylhydroperoxidase like protein, AhpD family
gene-VARPA_RS05935	11734.916	-2.921	0.452	-6.459	1.05E-10	3.17E-07	sorbitol/mannitol transport system substrate-binding protein
gene-VARPA_RS05940	1550.510	-3.007	0.481	-6.244	4.26E-10	8.46E-07	sorbitol/mannitol transport system permease protein

gene-VARPA_RS05945	1302.066	-1.744	0.427	-4.083	4.45E-05	0.005585192	sorbitol/mannitol transport system permease protein
gene-VARPA_RS05950	1880.259	-3.030	0.495	-6.124	9.12E-10	1.10E-06	multiple sugar transport system ATP-binding protein
gene-VARPA_RS05955	2129.811	-3.042	0.503	-6.047	1.48E-09	1.48E-06	D-arabinitol 4-dehydrogenase
gene-VARPA_RS05960	896.254	-2.701	0.411	-6.568	5.10E-11	3.07E-07	D-sorbitol dehydrogenase (acceptor)
gene-VARPA_RS06480	1705.775	-0.705	0.145	-4.874	1.10E-06	0.000499334	peptide/nickel transport system permease protein
gene-VARPA_RS06485	623.410	-0.700	0.206	-3.399	0.00067665	0.03884676	peptide/nickel transport system permease protein
gene-VARPA_RS06500	2706.780	-0.732	0.162	-4.508	6.55E-06	0.001643496	D-serine dehydratase
gene-VARPA_RS06505	1693.031	-0.734	0.191	-3.845	0.000120701	0.011604285	transcriptional regulator, RpiR family
gene-VARPA_RS06510	2424.563	-0.695	0.169	-4.119	3.81E-05	0.004940001	N-acyl-D-amino-acid deacylase
gene-VARPA_RS06580	2925.069	-0.912	0.238	-3.832	0.000127081	0.011604285	hypothetical protein
gene-VARPA_RS06640	39090.237	-4.345	1.134	-3.832	0.000127139	0.011604285	phenylacetate-CoA ligase
gene-VARPA_RS06645	12988.870	-4.675	1.259	-3.713	0.00020444	0.017848528	acyl-CoA thioesterase
gene-VARPA_RS06650	12767.400	-4.451	1.256	-3.544	0.000394517	0.027006465	2-(1,2-epoxy-1,2-dihydrophenyl)acetyl-CoA isomerase
gene-VARPA_RS06655	2343.276	-4.291	1.095	-3.919	8.88E-05	0.009065455	phenylacetic acid degradation protein
gene-VARPA_RS06660	3418.655	-3.560	0.813	-4.381	1.18E-05	0.00244377	regulatory protein TetR
gene-VARPA_RS06665	1363.065	-2.709	0.532	-5.093	3.53E-07	0.000194181	uncharacterized protein
gene-VARPA_RS06675	10478.697	0.420	0.124	3.376	0.000735406	0.0410193	Ca-activated chloride channel homolog
gene-VARPA_RS06850	19425.666	0.363	0.102	3.571	0.000355172	0.025741272	HNH endonuclease
gene-VARPA_RS06905	7450.647	-1.055	0.319	-3.313	0.000923222	0.048095594	large subunit ribosomal protein L33
gene-VARPA_RS08080	3108.448	0.716	0.172	4.150	3.33E-05	0.004640389	YCII-related protein
gene-VARPA_RS08085	3616.659	0.722	0.151	4.777	1.78E-06	0.000600232	YCII-related protein
gene-VARPA_RS08365	680.852	2.086	0.467	4.469	7.88E-06	0.001824862	short-chain dehydrogenase/reductase SDR
gene-VARPA_RS08700	806.255	0.418	0.126	3.321	0.00089682	0.047611647	iron(III) transport system permease protein
gene-VARPA_RS09940	834.856	0.855	0.243	3.520	0.000431748	0.029034786	hypothetical protein
gene-VARPA_RS09945	351.193	0.999	0.260	3.843	0.00012143	0.011604285	YqaJ viral recombinase family
gene-VARPA_RS09985	237.721	0.689	0.197	3.497	0.000471091	0.030846233	hypothetical protein
gene-VARPA_RS10025	188.919	0.726	0.210	3.458	0.000544421	0.032795934	hypothetical protein
gene-VARPA_RS10550	6226.248	-0.865	0.255	-3.399	0.00067711	0.03884676	long-chain acyl-CoA synthetase
gene-VARPA_RS11385	10387.955	-1.380	0.420	-3.283	0.001027125	0.04989841	fructose transport system permease protein
gene-VARPA_RS11390	11702.141	-1.266	0.365	-3.464	0.000531681	0.032352002	fructose transport system ATP-binding protein
gene-VARPA_RS11395	8079.355	-1.179	0.353	-3.337	0.000846471	0.046781095	ROK family protein
gene-VARPA_RS11490	1261.931	0.452	0.127	3.568	0.000359732	0.025741272	two-component system, OmpR family, response regulator
gene-VARPA_RS14250	4325.113	-1.268	0.276	-4.600	4.22E-06	0.001210646	GTP-binding protein

gene-VARPA_RS14255	540.305	-0.769	0.203	-3.783	0.000155043	0.01373495	protein of unknown function DUF6 transmembrane
gene-VARPA_RS15715	646.820	0.386	0.117	3.301	0.000962789	0.04873817	major facilitator superfamily MFS_1
gene-VARPA_RS16085	1190.303	-0.349	0.105	-3.322	0.000893211	0.047611647	HTH-type transcriptional regulator / antitoxin HipB
gene-VARPA_RS16240	3192.272	0.248	0.067	3.679	0.00023419	0.019869828	glutathione-dependent formaldehyde-activating GFA
gene-VARPA_RS17425	177.924	-0.827	0.230	-3.588	0.00033286	0.024675415	Acyl-CoA dehydrogenase type 2 domain
gene-VARPA_RS18845	967.364	1.069	0.289	3.699	0.000216472	0.018628936	monovalent cation:H+ antiporter, CPA1 family
gene-VARPA_RS18975	790.512	0.684	0.190	3.592	0.000328315	0.024675415	branched-chain amino acid transport system substrate-binding protein
gene-VARPA_RS19000	868.992	0.492	0.145	3.388	0.000703866	0.039626992	Choline dehydrogenase
gene-VARPA_RS19005	686.011	0.374	0.113	3.320	0.000901017	0.047611647	phenylacetaldehyde dehydrogenase
gene-VARPA_RS19030	691.310	0.474	0.143	3.325	0.000884439	0.047611647	hypothetical protein
gene-VARPA_RS19260	531.950	-1.598	0.459	-3.480	0.000501937	0.032166684	citrate lyase subunit beta / citryl-CoA lyase
gene-VARPA_RS20515	1593.926	0.338	0.102	3.310	0.000934128	0.048095594	MOSC domain containing protein
gene-VARPA_RS20600	2643.504	0.500	0.120	4.180	2.92E-05	0.004395705	transcriptional regulator, PadR-like family
gene-VARPA_RS21450	4642.810	0.628	0.181	3.467	0.000525924	0.03232822	methylated-DNA-[protein]-cysteine S-methyltransferase
gene-VARPA_RS21455	3453.376	0.719	0.167	4.308	1.65E-05	0.003012057	protein of unknown function DUF6 transmembrane
gene-VARPA_RS22075	1227.594	-1.510	0.383	-3.944	8.02E-05	0.008473819	choline dehydrogenase
gene-VARPA_RS22500	2844.348	-1.384	0.421	-3.286	0.001014476	0.04989841	general nucleoside transport system permease protein
gene-VARPA_RS22505	4069.625	-1.558	0.438	-3.553	0.000380912	0.026374874	general nucleoside transport system permease protein
gene-VARPA_RS22510	10758.387	-1.958	0.476	-4.116	3.85E-05	0.004940001	general nucleoside transport system ATP-binding protein
gene-VARPA_RS22515	32261.547	-4.348	0.921	-4.723	2.33E-06	0.000737482	putative MFS transporter, AGZA family, xanthine/uracil permease
gene-VARPA_RS22520	14008.160	-3.417	0.715	-4.775	1.79E-06	0.000600232	transcriptional regulator, LysR family
gene-VARPA_RS22525	39854.235	-3.473	0.952	-3.647	0.00026548	0.021323354	xanthine dehydrogenase large subunit
gene-VARPA_RS23070	4346.652	0.762	0.196	3.899	9.67E-05	0.009713017	DsrE family protein
gene-VARPA_RS24380	526.429	0.404	0.122	3.303	0.000956003	0.04873817	hypothetical protein
gene-VARPA_RS24805	21073.626	-1.384	0.421	-3.291	0.000997554	0.049663365	Peptidoglycan-binding lysin domain
gene-VARPA_RS24810	408.083	-0.939	0.261	-3.595	0.000324871	0.024675415	type III secretion protein U
gene-VARPA_RS24815	277.321	-1.051	0.245	-4.290	1.79E-05	0.003076042	type III secretion protein T
gene-VARPA_RS24820	342.009	-1.625	0.404	-4.018	5.87E-05	0.006796644	HrpD protein
gene-VARPA_RS24825	773.984	-1.699	0.397	-4.278	1.89E-05	0.003157215	ATP synthase in type III secretion protein N
gene-VARPA_RS24830	516.021	-1.535	0.355	-4.331	1.48E-05	0.002793683	type III secretion protein L
gene-VARPA_RS24835	416.597	-1.777	0.452	-3.934	8.36E-05	0.008686942	type III secretion protein K
gene-VARPA_RS24840	1320.693	-1.885	0.446	-4.229	2.34E-05	0.003714314	type III secretion protein J
gene-VARPA_RS24845	2060.699	-2.092	0.539	-3.881	0.000104061	0.010276433	hypothetical protein

gene-VARPA_RS24850	588.334	-2.024	0.463	-4.375	1.22E-05	0.00244377	Forkhead-associated protein
gene-VARPA_RS24855	791.848	-1.910	0.420	-4.550	5.36E-06	0.001467334	type III secretion protein C
gene-VARPA_RS24860	216.276	-2.333	0.543	-4.293	1.76E-05	0.003076042	type III secretion protein S
gene-VARPA_RS24865	754.204	-1.636	0.392	-4.171	3.03E-05	0.00444803	type III secretion protein V
gene-VARPA_RS24870	504.505	-1.585	0.382	-4.146	3.39E-05	0.004640389	type III secretion protein Q
gene-VARPA_RS24875	450.694	-1.303	0.291	-4.475	7.63E-06	0.001824862	type III secretion protein R
gene-VARPA_RS25645	1101.809	-1.736	0.413	-4.203	2.64E-05	0.004072624	hypothetical protein
gene-VARPA_RS25650	2887.327	-3.932	0.820	-4.797	1.61E-06	0.000600232	hypothetical protein
gene-VARPA_RS25655	1708.018	-4.509	0.886	-5.092	3.55E-07	0.000194181	hypothetical protein
gene-VARPA_RS25660	3220.042	-4.698	0.915	-5.132	2.86E-07	0.000191517	acetyl-CoA acyltransferase
gene-VARPA_RS25665	1607.546	-4.205	0.865	-4.862	1.16E-06	0.000499334	3-oxoadipate CoA-transferase, beta subunit
gene-VARPA_RS25670	829.156	-3.848	0.870	-4.423	9.73E-06	0.002092731	3-oxoadipate CoA-transferase, alpha subunit
gene-VARPA_RS26150	5272.600	-0.652	0.178	-3.659	0.000253644	0.020648016	LacI family transcriptional regulator
gene-VARPA_RS26155	3225.949	-0.519	0.143	-3.636	0.000277075	0.021676632	ribokinase
gene-VARPA_RS26385	930.593	0.312	0.091	3.421	0.000624692	0.036535401	phosphatidate cytidylyltransferase
gene-VARPA_RS26605	5428.829	0.454	0.134	3.392	0.000693903	0.039434664	DSBA oxidoreductase
gene-VARPA_RS27130	2493.563	0.393	0.099	3.974	7.06E-05	0.007734813	multiple antibiotic resistance (MarC)-related protein
gene-VARPA_RS27605	1958.584	-3.498	0.983	-3.558	0.000373183	0.026140183	type VI secretion system protein VasG
gene-VARPA_RS27625	2859.368	-3.905	1.123	-3.476	0.000508739	0.03225939	type VI secretion system secreted protein Hcp
gene-VARPA_RS27630	8540.904	-3.559	1.072	-3.321	0.00089794	0.047611647	type VI secretion system protein ImpC
gene-VARPA_RS27845	4119.352	-0.213	0.053	-4.047	5.19E-05	0.006301188	mannose PTS system EIIA component
gene-VARPA_RS28255	3305.549	0.356	0.089	4.006	6.17E-05	0.007008789	hypothetical protein
gene-VARPA_RS28625	4742.579	-1.346	0.340	-3.955	7.64E-05	0.008221609	ROK family protein
gene-VARPA_RS28630	7132.379	-1.376	0.396	-3.473	0.000515359	0.03232822	D-xylose transport system ATP-binding protein
gene-VARPA_RS29475	49591.126	-0.937	0.267	-3.513	0.000442761	0.029309789	type IV pilus assembly protein PilA
gene-VARPA_RS30095	1156.487	0.457	0.131	3.484	0.000493865	0.031989727	transcriptional regulator, AraC family
gene-VARPA_RS30350	3436.189	-2.010	0.579	-3.470	0.000520714	0.03232822	conserved membrane protein of unknown function
gene-VARPA_RS30885	3328.252	-0.729	0.212	-3.431	0.000601873	0.035545925	large subunit ribosomal protein L36
120 minutes							
gene-VARPA_RS00250	5671.207	0.426	0.125	3.407	0.000656369	0.039600387	tRNA uridine 5-carboxymethylaminomethyl modification enzyme
gene-VARPA_RS00260	2897.334	0.338	0.101	3.352	0.000801834	0.041640064	leucine efflux protein
gene-VARPA_RS00680	3469.517	-0.305	0.087	-3.483	0.000496415	0.036545467	hypothetical protein
gene-VARPA_RS00925	3569.012	1.552	0.456	3.401	0.00067126	0.039748631	transcriptional regulator, IclR family

gene-VARPA_RS00930	3947.929	1.593	0.406	3.926	8.65E-05	0.01841317	polar amino acid transport system substrate-binding protein
gene-VARPA_RS00935	1387.031	1.570	0.399	3.938	8.20E-05	0.01841317	polar amino acid transport system permease protein
gene-VARPA_RS00955	2304.584	1.523	0.438	3.474	0.000511992	0.036960452	formimidoylglutamate deiminase
gene-VARPA_RS00960	1875.143	1.550	0.413	3.754	0.000173764	0.020524602	N-formylglutamate deformylase
gene-VARPA_RS01000	12389.710	0.440	0.106	4.156	3.23E-05	0.018095676	5-methyltetrahydrofolate--homocysteine methyltransferase
gene-VARPA_RS01295	5981.375	0.607	0.164	3.700	0.000215786	0.023088317	recombination associated protein RdgC
gene-VARPA_RS01305	5752.472	0.494	0.145	3.400	0.000673035	0.039748631	Zinc finger, CHCC-type
gene-VARPA_RS01355	1453.980	0.433	0.122	3.550	0.000385867	0.032738948	peptidase M48 Ste24p
gene-VARPA_RS01440	1320.357	0.512	0.152	3.362	0.000773223	0.040503454	exodeoxyribonuclease III
gene-VARPA_RS01580	28553.155	-3.541	1.048	-3.381	0.00072264	0.040503454	putative spermidine/putrescine transport system substrate-binding protein
gene-VARPA_RS01905	3187.362	-2.308	0.464	-4.977	6.46E-07	0.00129775	Methyltransferase type 12
gene-VARPA_RS01995	4588.638	0.793	0.217	3.653	0.000259588	0.025474185	single-strand DNA-binding protein
gene-VARPA_RS03125	5240.920	2.188	0.581	3.766	0.000165844	0.020388707	anthranilate synthase component I
gene-VARPA_RS03135	3582.229	2.032	0.548	3.707	0.000209423	0.022937557	anthranilate synthase component II
gene-VARPA_RS03140	4899.548	2.165	0.556	3.892	9.94E-05	0.01841317	threonine aldolase
gene-VARPA_RS03145	1690.715	1.994	0.524	3.807	0.000140844	0.018938281	Lysine exporter protein (LYSE/YGGA)
gene-VARPA_RS03150	4870.499	2.049	0.538	3.806	0.000141471	0.018938281	anthranilate phosphoribosyltransferase
gene-VARPA_RS03155	3947.615	1.860	0.509	3.650	0.000262185	0.025474185	indole-3-glycerol phosphate synthase
gene-VARPA_RS03160	2137.350	1.915	0.476	4.027	5.64E-05	0.01841317	uracil-DNA glycosylase
gene-VARPA_RS03180	39509.623	1.176	0.311	3.777	0.000158978	0.020376243	elongation factor Tu
gene-VARPA_RS03560	3515.203	-0.430	0.109	-3.935	8.33E-05	0.01841317	hyalin repeat-containing protein
gene-VARPA_RS03635	479.446	0.382	0.111	3.432	0.000599241	0.037998202	alkyl hydroperoxide reductase/Thiol specific antioxidant/Mal allergen
gene-VARPA_RS03770	1077.194	0.977	0.291	3.364	0.000768087	0.040503454	short-chain dehydrogenase/reductase SDR
gene-VARPA_RS04170	6625.115	0.658	0.198	3.319	0.000902638	0.045604078	Protein of unknown function DUF2147
gene-VARPA_RS04515	3724.554	0.799	0.237	3.368	0.000756268	0.040503454	cysteine synthase
gene-VARPA_RS04530	338.711	0.660	0.195	3.378	0.000729258	0.040503454	MarR family transcriptional regulator, transcriptional regulator for hemolysin
gene-VARPA_RS05075	1183.661	0.397	0.115	3.465	0.000530147	0.037571863	cytochrome c assembly protein
gene-VARPA_RS05080	3024.115	0.614	0.160	3.833	0.000126352	0.018564534	signal recognition particle subunit SRP54
gene-VARPA_RS06385	22193.737	0.979	0.277	3.531	0.000413364	0.033201403	ubiquinol-cytochrome c reductase cytochrome b subunit
gene-VARPA_RS06395	8247.367	1.096	0.319	3.436	0.000591159	0.037998202	stringent starvation protein A
gene-VARPA_RS07155	972.260	-1.044	0.303	-3.445	0.000570029	0.037998202	alpha-L-fucosidase 2
gene-VARPA_RS07645	11398.889	0.302	0.087	3.456	0.000549073	0.037998202	large subunit ribosomal protein L32
gene-VARPA_RS08005	5582.128	-0.403	0.118	-3.411	0.000646787	0.039600387	salicylate hydroxylase

gene-VARPA_RS08060	7224.983	0.652	0.173	3.766	0.000165724	0.020388707	small subunit ribosomal protein S20
gene-VARPA_RS08140	3108.496	0.398	0.082	4.867	1.14E-06	0.001710047	uncharacterized protein
gene-VARPA_RS08160	5967.519	1.031	0.260	3.965	7.34E-05	0.01841317	small subunit ribosomal protein S16
gene-VARPA_RS08510	5748.445	1.090	0.329	3.315	0.000917141	0.045604078	carboxymethylenebutenolidase
gene-VARPA_RS08900	2340.931	0.374	0.113	3.313	0.000923589	0.045604078	lipopolysaccharide assembly protein B
gene-VARPA_RS08905	613.148	0.481	0.142	3.375	0.000736966	0.040503454	lipopolysaccharide assembly protein A
gene-VARPA_RS09805	3182.530	0.873	0.207	4.206	2.59E-05	0.018095676	elongation factor P
gene-VARPA_RS09865	6445.944	0.654	0.194	3.373	0.000744335	0.040503454	peptidyl-prolyl cis-trans isomerase D
gene-VARPA_RS10340	1331.195	1.466	0.376	3.900	9.60E-05	0.01841317	catecholate siderophore receptor
gene-VARPA_RS10355	765.170	2.067	0.542	3.813	0.000137243	0.018938281	Nickel transport complex, NikM subunit, transmembrane
gene-VARPA_RS10360	296.927	1.440	0.399	3.606	0.00031058	0.02834748	hypothetical protein
gene-VARPA_RS10435	6983.223	1.214	0.371	3.274	0.001061566	0.049297929	primosomal replication protein N
gene-VARPA_RS10440	7089.342	1.353	0.382	3.539	0.000401959	0.033169905	small subunit ribosomal protein S18
gene-VARPA_RS10500	3944.153	0.895	0.233	3.840	0.000123274	0.018564534	homoserine dehydrogenase
gene-VARPA_RS10780	4550.223	0.528	0.154	3.439	0.000583733	0.037998202	acetyl-CoA acyltransferase
gene-VARPA_RS10790	1870.440	0.414	0.108	3.846	0.000120305	0.018564534	fluoroquinolone resistance protein
gene-VARPA_RS10950	8636.689	0.392	0.110	3.565	0.000363673	0.031652399	4-hydroxy-tetrahydrodipicolinate synthase
gene-VARPA_RS11860	1960.928	-1.363	0.411	-3.315	0.000918005	0.045604078	hypothetical protein
gene-VARPA_RS11970	18275.923	0.940	0.278	3.379	0.000727027	0.040503454	acyl-CoA dehydrogenase domain-containing protein
gene-VARPA_RS11980	27798.263	0.872	0.253	3.444	0.000573069	0.037998202	3-hydroxyisobutyrate dehydrogenase
gene-VARPA_RS11995	1221.507	0.352	0.099	3.564	0.000365465	0.031652399	LysR family transcriptional regulator, glycine cleavage system transcriptional activator
gene-VARPA_RS12150	2206.299	0.482	0.135	3.562	0.000367807	0.031652399	hypothetical protein
gene-VARPA_RS12515	5622.879	0.797	0.204	3.916	9.00E-05	0.01841317	large subunit ribosomal protein L31
gene-VARPA_RS12805	4271.673	0.818	0.210	3.889	0.000100754	0.01841317	2-isopropylmalate synthase
gene-VARPA_RS13405	311.024	-0.425	0.115	-3.677	0.000235712	0.024066596	3-hydroxypropanoate dehydrogenase
gene-VARPA_RS13585	3819.867	-0.388	0.105	-3.713	0.000204952	0.022863545	23S rRNA (pseudouridine1915-N3)-methyltransferase
gene-VARPA_RS13805	17011.690	-0.648	0.171	-3.788	0.000151733	0.019870366	hypothetical protein
gene-VARPA_RS14120	2774.213	0.380	0.108	3.527	0.000419896	0.033282315	lipopolysaccharide export system permease protein
gene-VARPA_RS14455	479.884	-0.446	0.121	-3.681	0.000232623	0.024066596	type VI secretion system secreted protein VgrG
gene-VARPA_RS14615	661.159	-0.426	0.128	-3.335	0.000853413	0.043567472	Pirin domain protein
gene-VARPA_RS16145	3643.932	0.446	0.122	3.664	0.00024877	0.024976518	acyl carrier protein
gene-VARPA_RS16355	2136.841	0.572	0.174	3.285	0.001018805	0.049098259	uncharacterized protein
gene-VARPA_RS16485	1595.687	0.802	0.220	3.644	0.000268884	0.0257104	hypothetical protein

gene-VARPA_RS16490	1639.155	0.844	0.196	4.316	1.59E-05	0.015976451	transcription elongation factor GreA
gene-VARPA_RS16505	4958.413	0.630	0.173	3.634	0.000278794	0.026241491	carbamoyl-phosphate synthase small subunit
gene-VARPA_RS17095	10451.843	0.795	0.212	3.746	0.000179364	0.020778578	trigger factor
gene-VARPA_RS17375	38.448	-1.320	0.344	-3.836	0.000124883	0.018564534	branched-chain amino acid transport system ATP-binding protein
gene-VARPA_RS17515	2662.431	0.368	0.105	3.517	0.000436752	0.033730709	Tetratricopeptide TPR_1 repeat-containing protein
gene-VARPA_RS17520	3562.216	0.889	0.221	4.026	5.67E-05	0.01841317	peptidyl-prolyl cis-trans isomerase A (cyclophilin A)
gene-VARPA_RS18565	16683.133	1.701	0.436	3.901	9.59E-05	0.01841317	propionyl-CoA synthetase
gene-VARPA_RS18750	1403.817	1.697	0.411	4.127	3.68E-05	0.018095676	Lrp/AsnC family transcriptional regulator, leucine-responsive regulatory protein
gene-VARPA_RS18755	6152.104	2.014	0.536	3.755	0.000173287	0.020524602	lactoylglutathione lyase
gene-VARPA_RS18760	13364.411	2.061	0.553	3.726	0.000194329	0.022087461	biotin synthase
gene-VARPA_RS18765	26154.523	2.310	0.596	3.876	0.000106303	0.01841317	propionyl-CoA carboxylase alpha chain
gene-VARPA_RS18770	21283.097	2.358	0.589	4.001	6.30E-05	0.01841317	propionyl-CoA carboxylase beta chain
gene-VARPA_RS18780	4055.276	1.874	0.517	3.625	0.000289243	0.026806107	GCN5-related N-acetyltransferase
gene-VARPA_RS18785	20143.724	2.035	0.530	3.836	0.00012517	0.018564534	methylmalonyl-CoA mutase
gene-VARPA_RS18790	5279.043	1.582	0.473	3.342	0.000830469	0.042758514	transcriptional regulator, GntR family
gene-VARPA_RS20190	1208.916	0.378	0.110	3.439	0.000582966	0.037998202	transposase
gene-VARPA_RS20245	11281.138	0.452	0.117	3.866	0.000110476	0.018486313	small subunit ribosomal protein S21
gene-VARPA_RS20250	2073.268	0.674	0.190	3.541	0.00039904	0.033169905	uncharacterized protein
gene-VARPA_RS20255	1322.971	0.885	0.251	3.521	0.000430423	0.033673589	NUDIX hydrolase
gene-VARPA_RS21210	7112.715	0.756	0.191	3.950	7.81E-05	0.01841317	energy-dependent translational throttle protein EttA
gene-VARPA_RS21320	566.938	1.071	0.307	3.490	0.000483346	0.036395948	glutathione S-transferase
gene-VARPA_RS21400	1340.430	0.577	0.176	3.273	0.001063866	0.049297929	hypothetical protein
gene-VARPA_RS21405	2144.445	0.598	0.119	5.016	5.28E-07	0.00129775	peptide-methionine (S)-S-oxide reductase
gene-VARPA_RS21545	595.675	-0.384	0.114	-3.381	0.00072224	0.040503454	hypothetical protein
gene-VARPA_RS22025	4094.334	-0.524	0.135	-3.878	0.000105396	0.01841317	long-chain acyl-CoA synthetase
gene-VARPA_RS22540	1496.716	-2.063	0.540	-3.818	0.000134392	0.018938281	rhamnosyltransferase
gene-VARPA_RS22550	3174.845	-2.387	0.710	-3.363	0.000772289	0.040503454	glycosyltransferase, MGT family
gene-VARPA_RS22555	17986.560	-2.438	0.659	-3.697	0.000218465	0.023088317	nonribosomal peptide synthetase DhbF
gene-VARPA_RS23245	1027.770	0.418	0.105	3.971	7.17E-05	0.01841317	polyphosphate kinase
gene-VARPA_RS23705	7766.219	0.495	0.144	3.434	0.000593855	0.037998202	methionyl-tRNA synthetase
gene-VARPA_RS23710	778.688	0.963	0.248	3.874	0.000106982	0.01841317	hypothetical protein
gene-VARPA_RS24750	4270.735	0.694	0.154	4.508	6.56E-06	0.00789939	domain of unknown function DUF1732
gene-VARPA_RS24950	4208.774	1.063	0.301	3.533	0.000411438	0.033201403	ribose-phosphate pyrophosphokinase

gene-VARPA_RS25810	7657.067	0.544	0.162	3.366	0.000761813	0.040503454	cytochrome c class I
gene-VARPA_RS26105	1061.506	0.417	0.120	3.482	0.000497465	0.036545467	hypothetical protein
gene-VARPA_RS26165	2792.477	0.297	0.073	4.083	4.45E-05	0.01841317	uncharacterized protein
gene-VARPA_RS26440	13063.275	0.706	0.201	3.505	0.000457272	0.034868409	Peptidase M23
gene-VARPA_RS26895	8761.157	0.608	0.178	3.407	0.000657377	0.039600387	glycyl-tRNA synthetase beta chain
gene-VARPA_RS26900	3057.707	0.502	0.100	5.007	5.52E-07	0.00129775	glycyl-tRNA synthetase alpha chain
gene-VARPA_RS26920	20462.913	1.798	0.527	3.414	0.000640493	0.039600387	glutamin-(asparagin-)ase
gene-VARPA_RS27305	24510.077	1.800	0.549	3.281	0.001034752	0.049297929	glutamate dehydrogenase (NAD(P)+)
gene-VARPA_RS27530	8019.405	1.308	0.396	3.303	0.000957528	0.046517335	F-type H+-transporting ATPase subunit c
gene-VARPA_RS27705	1438.871	-0.510	0.149	-3.427	0.000610118	0.038284897	transmembrane protein
gene-VARPA_RS27960	11590.057	1.108	0.285	3.887	0.000101413	0.01841317	small subunit ribosomal protein S12
gene-VARPA_RS28710	31727.223	-3.219	0.956	-3.366	0.000762427	0.040503454	Endoribonuclease L-PSP
gene-VARPA_RS28715	21230.041	-1.771	0.439	-4.036	5.44E-05	0.01841317	transcriptional regulator, AraC family
gene-VARPA_RS28720	677.022	-0.756	0.223	-3.386	0.000709164	0.040503454	metal-dependent phosphohydrolase HD sub domain
gene-VARPA_RS28790	8960.402	1.194	0.346	3.450	0.000559914	0.037998202	small subunit ribosomal protein S14
gene-VARPA_RS28795	12543.234	1.313	0.319	4.113	3.91E-05	0.018095676	large subunit ribosomal protein L5
gene-VARPA_RS28800	7450.631	1.276	0.307	4.150	3.33E-05	0.018095676	large subunit ribosomal protein L24
gene-VARPA_RS28805	7858.936	1.364	0.393	3.473	0.000515385	0.036960452	large subunit ribosomal protein L14
gene-VARPA_RS29145	2914.442	1.035	0.316	3.274	0.001059183	0.049297929	preprotein translocase subunit YajC
gene-VARPA_RS29510	17926.614	0.867	0.262	3.309	0.000936337	0.04585766	succinyl-CoA synthetase beta subunit
gene-VARPA_RS29615	851.883	-0.421	0.129	-3.273	0.001062706	0.049297929	major facilitator superfamily MFS_1
gene-VARPA_RS30040	3004.709	0.986	0.239	4.118	3.83E-05	0.018095676	ribonuclease P protein component
gene-VARPA_RS30550	646.157	0.593	0.152	3.911	9.20E-05	0.01841317	bacterioferritin-associated ferredoxin
gene-VARPA_RS30710	10927.675	-0.806	0.234	-3.445	0.000570222	0.037998202	hypothetical protein
gene-VARPA_RS31390	5949.558	2.064	0.528	3.912	9.14E-05	0.01841317	LAO/AO transport system kinase
