# Supplementary material for: Rhizospheric miRNAs affect the plant microbiota
Source: ISME Commun. 2024 Oct 12;4(1):ycae120. doi: 10.1093/ismeco/ycae120 (PMC11520407; doi:10.1093/ismeco/ycae120)
Supplement: SupplementaryTableS2_ycae120 [file supplementarytables2_ycae120.docx]

| **miRNA** | **Sequences** |
| --- | --- |
| ath-miR158a-3p | UCCCAAAUGUAGACAAAGCA |
| ath-miR158b | CCCCAAAUGUAGACAAAGCA |
| ath-miR159a | UUUGGAUUGAAGGGAGCUCUA |
| ath-miR159b-3p | UUUGGAUUGAAGGGAGCUCUU |
| ath-miR159c | UUUGGAUUGAAGGGAGCUCCU |
| ath-miR161.1 | UGAAAGUGACUACAUCGGGGU |
| ath-miR165b | UCGGACCAGGCUUCAUCCCCC |
| ath-miR827 | UUAGAUGACCAUCAACAAACU |
| ath-miR5642b | UCUCGCGCUUGUACGGCUUU |
| sc-ath-miR158a-3p | CAACAAGAUGACAAUGUCAC  UGAAAACAUACAUCAGACCG* |
| sc-ath-miR158b | GACAAGCUCCACAUACAGAA |
| sc-ath-miR159a | AGGGAAGUAUUGUCGUGACUU  AGAGAGACCUUGGGUUGAUUU** |
| sc-ath-miR159b-3p | UGGGGACUGUAUCUUAGUUAG |
| sc-ath-miR159c | CAGGCCUUGGUGUUAUAUGAG |
| sc-ath-miR161.1 | CUGCAGCUAGUUGGGUAAAGA |
| sc-ath-miR165b | CUCCACCGAGGUCCUCCACGU |
| sc-ath-miR827 | GAUCCAAAAAGCUAAUUUCAC |
| sc-ath-miR5642b | ACGUCGUAAAACUUCACGUG |

**Supplementary Table S2**: Sequences of the single-stranded synthetic miRNAs.

* and **: Scramble miRNAs used in the simplified soil community experiment

** Scramble miRNA ath-miR159a used during confocal microscopy imaging and flow cytometry. Note: all sequences were methylated at their 3’ end.
