# Supplementary material for: Rhizospheric miRNAs affect the plant microbiota
Source: ISME Commun. 2024 Oct 12;4(1):ycae120. doi: 10.1093/ismeco/ycae120 (PMC11520407; doi:10.1093/ismeco/ycae120)
Supplement: SupplementaryTableS3_ycae120 [file supplementarytables3_ycae120.docx]

**Supplementary Table S3**: Primers used for qPCR in *Arabidopsis thaliana* and *Variovorax paradoxus* EPS for the *in vitro* miPEP transcriptomic experiment. Most primers were designed based on the EPS genome, except for plant gene primers.


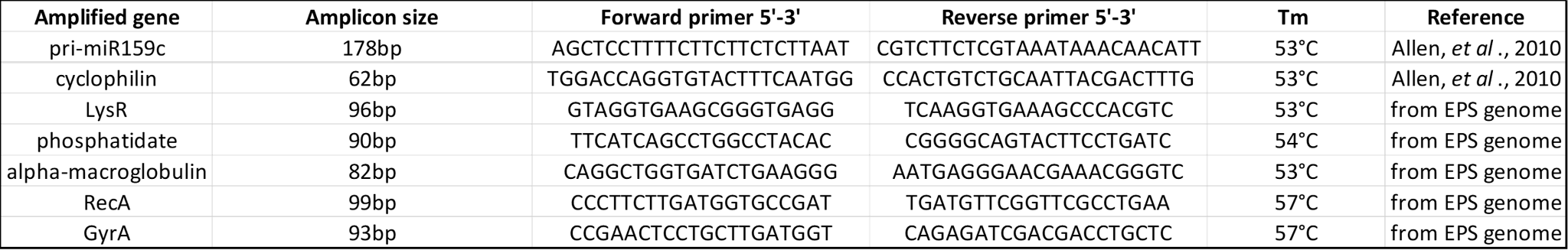


Allen R. S., Li J., Alonso-Peral M. M., White R. G., Gubler F., Millar A. A. (2010). MicroR159 regulation of most conserved targets in Arabidopsis has negligible phenotypic effects. Silence 1:18. doi: 10.1186/1758-907X-1-18
