# Supplementary material for: Rhizospheric miRNAs affect the plant microbiota
Source: ISME Commun. 2024 Oct 12;4(1):ycae120. doi: 10.1093/ismeco/ycae120 (PMC11520407; doi:10.1093/ismeco/ycae120)
Supplement: 20240510SuppMethods_ycae120 [file 20240510suppmethods_ycae120.docx]

**Supplementary methods for:**

**Rhizospheric miRNAs affect the plant microbiota.**

Harriet Middleton^1,2^#, Jessica Dozois^2^#, Cécile Monard^1^, Virginie Daburon^1^, Emmanuel Clostres^1^, Julien Tremblay^2^, Jean-Philippe Combier^3^, Étienne Yergeau^2^* and Abdelhak El Amrani^1^*

^1^ECOBIO, CNRS UMR 6553, Université de Rennes, Campus Beaulieu, Rennes, France

^2^Institut National de la Recherche Scientifique, Centre Armand-Frappier Santé Biotechnologie, Laval, Québec, Canada.

^3^ LRSV, UMR CNRS/UT3/INP 5546, Auzeville-Tolosane, France.

#Contributed equally.

*Corresponding authors: [abdelhak.elamrani@univ-rennes1.fr](mailto:abdelhak.elamrani@univ-rennes1.fr) and [Etienne.Yergeau@inrs.ca](mailto:Etienne.Yergeau@inrs.ca)

**Detection of plant miRNAs in the roots and the rhizosphere, and associated with rhizosphere bacteria**

*Plant growth conditions.*

For the detection of miRNA in the rhizosphere, *Arabidopsis thaliana* (Col-0) and *Brachypodium distachyon* (Bd21-3) were planted in triplicates and grown for a month in a growth cabinet, alongside unplanted soils. For the detection of miRNA in roots, *Arabidopsis thaliana* (Col-0) was planted in ten different experimental blocks and grown for 21 days in a growth cabinet, alongside unplanted soils. The program of the growth cabinets was: 12 hours of daylight, 3 hours of twilight, 3 hours of dawn light, and 6 hours of darkness at a constant humidity of 70%. The temperature varied in accordance with the light cycle between 20-25 °C.

*Sampling, RNA extraction and sequencing*

The rhizospheres and control soils were sampled and flash-frozen in liquid nitrogen. RNA was then extracted from 2 g of soil using MOBIO RNA Power soil kit, eluted in 100 µL, following the manufacturer’s guidelines. The roots were gently and quickly washed with sterile PBS, placed in a sterile tube and flash frozen with liquid nitrogen. The roots were lyophilized for three hours, crushed with a micro pestle, and RNA was extracted using the Qiagen RNeasy Plant Mini kit, following the manufacturer’s guidelines. The RNA was treated with DNase (Turbo DNA-free kit, Ambion), purified and concentrated (RNA Clean & Concentrator 25μl, ZymoResearch). We evaluated RNA quality with Nanodrop and 4% agarose gels, before sending it for sequencing on a HiSeq 2500 system (Illumina) in single read mode (1x50bp) at the Centre d’expertise et de services Génome Québec (Montreal, Canada).

*Bioinformatic analyses*

Reads were trimmed using Trimmomatic [1] (v0.39) to remove the sequencing adapters with the following Illumina clip settings :5:10:4 and to remove the 5’ and 3’ flanking sequences containing bases having quality scores below 33 and 30, respectively. Common contaminants, such as Illumina adapters and PhiX spike-in sequences, were removed using BBDuk (BBMap v38.11, <https://github.com/BioInfoTools/BBMap>), with key parameters: k=20 and minkmerhits=1. Remaining sequences were then filtered by size such that reads having a length between 18 and 27 nucleotides were selected. Selected reads were mapped against either *A. thaliana* (TAIR10/GCA_000001735.1) or *B. distachyon* (bd21/GCF_000005505.1) reference genomes, which are the same as those used in miRbase [2], using BWA-aln [3] with the following parameters: mismatch=1 and seed=5. Using BEDTools v.2.29.2 [4] with -F 0.95 argument, a number of reads aligned inside miRNA coordinates were determined, meaning that at least 95% of each read had to belong inside the miRNA coordinates to be considered a valid hit. These assigned miRNAs which were mapped against the reference database were kept for abundance estimation across samples.

*Statistical analyses*

Rhizospheric and root miRNAs were defined as the miRNAs that had at least 10 reads for each the rhizosphere or root samples and a maximum of one read across all the bulk soil samples.

*Detection of plant miRNAs in or on rhizospheric bacteria*

Plant growth and bacteria isolation. *A. thaliana* Col-0 was grown for a month, then the rhizosphere was sampled. Bacteria were isolated from 5 g of rhizospheric soil, using a density gradient. Samples were added to 30 mL of 0.2% sodium hexametaphosphate and rotated on a spinning wheel for 2 h. Samples were then centrifuged at 18 *g* for 1 min at 10°C, the supernatants were pooled two-by-two and centrifuged at 2824 x *g* for 20 min at 10°C. The microbe-containing pellet was resuspended in 10 mL of 0.8% NaCl and samples were again pooled by two. The resuspended solution was then transferred onto 10 mL of sterile Nycodenz and was centrifuged at 3220 *g* for 40 min. The bacterial layer was collected and a volume of 0.8% NaCl sufficient for a total of 35 mL was added. This solution was centrifuged at 3220 *g* for 15 min at 10°C. Finally, the pellet was resuspended in 1 mL of NaCl 0.8% to wash the residual Nycodenz away and centrifuged at 1700 *g* for 5 min at 10°C. The final pellet was resuspended in 100 µL TE buffer and kept at -20°C. Eight bacterial samples were obtained from the rhizosphere and six from unplanted soils.

RNA extraction and sequencing. RNA was extracted from this bacterial solution as follows: the 100 µL were transferred into a MP Biomedicals™ Lysing Matrix E tube with 250 µL of warmed 10% CTAB (Cetyl Trimethyl Ammonium Bromide) - 0.7 M NaCl, followed by 250 µL of 240 mM K_2_HPO_4_/KH_2_PO_4_ (pH 8.0) and 500 µL of phenol-chloroform-isoamyl alcohol (25:24:1) (pH 8.0). Samples were then bead-beaten using TissueLyser II (QIAGEN) at 30 Hz for 3 min. The tubes were centrifuged at 16000 *g* for 10 min at 4°C and the supernatant was transferred into a new tube. One volume of chloroform-isoamyl alcohol (24:1) was added to the supernatant and centrifuged again at 16,000 *g* for 10 min at 4°C. The supernatant was collected and 2 volumes of 30% PEG 6000-1.6 M NaCl were added, and the tube was inverted to mix and was left at 4°C for at least 2 h. The tubes were then centrifuged at 18,000 *g* for 30 min at 4°C to precipitate RNA. The RNA pellet was then washed with 500 µL of 70% ethanol and centrifuged at 18,000 *g* for 10 min at 4°C. The pellet was then air dried and resuspended in 50 µL of RNAse-free water. RNA quality and quantity was verified using a 1% agarose gel and Nanodrop, and then sent for sequencing on a NovaSeq 6000 (Illumina) using paired-end 100 bp with size-selected library.

Bioinformatic analyses. Sequencing results were processed as stated above with the rhizospheric miRNAs. To confirm that miRNAs found in the rhizospheric bacteria originated from the plant and not the bacteria, miRNA sequences were searched for on the + and - strands of 3,837 bacterial genomes [5], of which 1,160 bacteria were isolated from plants. These miRNA sequences were also searched for in the genome of *A. thaliana* TAIR10.1 in which they were found at their expected position.

Statistical analyses. We used the same strategy as above to define the internalized rhizospheric miRNAs, but with a lower threshold because there were less plant miRNA reads produced for this experiment. We defined the internalized miRNAs as the miRNAs that were represented by at least 5 reads in each rhizosphere microbial pellets and absent in the bulk soil microbial pellets.

**Effect of miRNAs on bacterial gene expression**

*In silico analysis of miRNA targets*

To predict potential plant miRNA targets in bacterial genomes, we implemented a workflow named *mirnatarget 1.0 : miRNA target finder* (https://github.com/jtremblay/MiRNATarget), which was inspired from the plant miRNA target finder, *psRNAtarget* [6]. This tool is based on specific pairing patterns between plant miRNA and targets [7] and implements SSEARCH36 (from the fasta36 v36.3.8 package) which uses the Smith-Waterman local alignment algorithm, resulting in optimal alignments [8]. Multiple targets for each miRNA can be identified. Based on the *psRNAtarget* rules for miRNA-target recognition, an *e*-value was calculated for each alignment and only those with an *e*-value ≤ 5 were retained. The positions of the targeted regions were noted with respect to the neighbouring coding sequences (CDS): the target sequence could be inside a CDS, at the 5’ or 3’ flanking regions (FLR) or in other short/very short inter-CDS regions, overlapping CDSs or elsewhere, further away from any CDS. Targets in the latter category were positioned at a distance superior to 100 nt from the 5’ end or at a distance superior to 350 nt from the 3’ end of the CDS. These distances were chosen as they correspond to the longest UTRs that are implicated in gene regulation. Targets in this category were discarded from the analysis, because it was considered that a miRNA in that area would not affect mRNA translation. The annotation of targeted regions and extraction of the closest CDSs were carried out with our tool “align2cdsRegions” (<https://github.com/ntzv-git/align2cdsRegions>). Sequences of targeted CDSs were retrieved using the *getfasta* tool from BEDTools [4] (v2.30.0) and GO (Gene Ontology) terms were attributed using InterProScan (v5.59-91.0) [9]. For each domain of GO (*i.e*. Biological Processes, Cellular Component, Molecular Function) and for each targeted CDS, a GO ancestor and their description were associated, and a level of hierarchical ontology was attributed [10].

*Bacterial transcriptomic response to miRNAs*

Bacterial growth and miRNA treatment. *Variovorax paradoxus* EPS was grown in 5% yeast extract (YE), at 30°C with 250 rpm shaking. *Bacillus mycoides* YL123 was grown in tryptic soy broth (TSB), at 25°C with 250 rpm shaking. Bacterial stocks were plated on YE or TSB and single colonies were selected for liquid culture overnight (*n* = 3), in 20 mL YE or TSB. In the morning, cultures were normalised as such: the optical density (OD) of overnight cultures was measured at 600 nm, which were then centrifuged at 5000 rpm for 5 min to collect the bacterial pellet. The supernatant was removed, and a calculated volume of medium was added to the pellet to reach OD = 1 for *B. mycoides* (*i.e.* 7.36 x 10^8^CFU/mL) and OD = 0.3 for *V. paradoxus* (*i.e.* 9.99 x 10^8^CFU/mL). Bacterial cultures were left to grow until the exponential phase was reached, which was determined previously (4-5 h for both strains). OD was then measured to check that the maximum number of cells/mL for RNA extraction was not exceeded (10^9^ cells/mL). At this point, 1 µg of the miRNA mix, or scrambled mix was applied to the cultures and gently mixed (15 µL of each miRNA solution diluted at 10 µM). Cultures were normalised to reach the same number of bacterial cells per mL. The volume of the cultures was varied to mimic natural differences in rhizosphere volume. Total concentration of miRNAs ranged between 65 nM-104 nM for *B. mycoides* and 45 nM-85 nM for *V. paradoxus*. The same cultures were sampled after 20 min and 120 min of incubation, to evaluate the evolution of gene expression in a same biological sample in response to a short and long confrontation with miRNAs. The miRNA mix was composed of 6 rhizospheric miRNAs : miR159a, miR159b, miR159c, miR161.1, miR158a and miR165b (Supplementary Table S2). The scrambled mix was composed of 6 RNA sequences with the same nucleic acids as the respective miRNAs but in a random order (Supplementary Table S2). To resemble mature plant miRNAs found in the rhizosphere, these synthetic miRNAs were single-stranded with a 3’ methyl group. They were synthesised by Integrated DNA Technologies (IDT).

RNA extraction. After incubation, 1 mL of every culture was sampled and centrifuged at 5000 rpm for 5 min. The bacterial pellet was resuspended in 100 µL of a lysozyme solution: 1 mg/mL for *V. paradoxus* (Gram -) and 3 mg/mL for *B. mycoides* (Gram +). After vortexing the resuspension, the samples were left to incubate for 15 min at room temperature. The following steps used solutions from the RNeasy Plant Mini Kit (QIAGEN). The guanidine-thiocyanate-based buffer RLT was added to the samples for further lysis (600 µL) and vortexed for 10 s. For *B. mycoides* samples, an additional step was necessary: the 700 µL were transferred to a sterile 2 mL Safe-Lock tube containing 100 mg glass beads (0.1 mm diameter) and 100 mg zirconia/silica beads (0.5 mm diameter). Beads were resuspended by pipetting then cells were disrupted using Fast Prep for 50 s (5 m/s), followed by 5 min on ice and another 50 s of Fast Prep. Tubes were centrifuged at 10 000 *g* for 1 min and supernatant was transferred to a new tube. The next steps were common to both strains. An equal volume of 70% ethanol was added to the lysed samples and mixed by pipetting. The lysate was transferred to a RNeasy spin column (pink column) in a 2 mL collection tube and centrifuged at 10 000 rpm for 30 s, then the flow-through was discarded. Columns were first washed with 700 µL of the RW1 wash buffer, followed by 500 µL RPE buffer and again with 500 µL RPE buffer. Collection tube was changed, and the column was centrifuged for 1 min at maximum speed to dry the membrane off. To elute RNA, columns were placed in new 1.5 mL tubes and 30 µL of RNAse-free water was added before centrifugation. The 30 µL were then reloaded and centrifuged one last time.

RNA-seq & sequence analysis. Total RNA was sent for sequencing at the Centre d’expertise et de services Génome Québec (Montreal, Canada), where ribosomal RNAs were depleted and messenger RNAs were sequenced. RNA-seq raw reads were processed with ShotgunMG [11]. Reads were trimmed and filtered for quality (Trimmomatic v.39) and mapped (BWA v0.7.17) against their respective reference genome. The genome of *B. mycoides* YL123 originated from our lab, was sequenced, processed and submitted to the NCBI through the Prokaryotic Genome Annotation Pipeline (PGAP) under the assembly accession GCA_024297165.1 on GenBank. The *V. paradoxus* EPS genome was published with accession number NC_014931 [12]. BedTools v2.23.0 was used to generate read count files from each .bam file that were then merged to generate a raw count matrix. The latter file was used as input data for a differential gene expression analysis using DESEq2 (Bioconductor, version 1.36.0). For each strain and each time point, gene expression of samples treated with synthetic miRNAs was compared to those treated with scrambled synthetic miRNAs.

*Confocal microscopy.*

*Variovorax paradoxus* EPS and *Bacillus mycoides* YL123 were cultured on solid media (Bacto Yeast Extract Agar for *V. paradoxus* EPS and Tryptic Soy Agar for *B. mycoides* YL123) for 48 hours and then a few colonies were inoculated in 5 mL of the equivalent liquid media and grown overnight (250 rpm 28 ºC). The cultures were then standardized to an OD600 of 0.200, treated with one of the 3’-Cy5-labelled oligos (plant ath-miR159a-Cy5, scrambled ath-miR159a-Cy5 or a modified version of cytidine pCp-Cy5, final concentration 2 µM) (Supplementary Table S2) and incubated for a total of 3 hours and 40 minutes (the cultures were within the log phase) after which they were treated with MitoTracker Green FM (Invitrogen, final concentration 2 μM). The cultures were immediately reincubated for 20 minutes, pelleted, washed with sterile PBS, pelleted again, and concentrated 10 times in sterile PBS. The cells (4.5 μl of washed and concentrated culture) were then placed on a glass bottom dish, covered with a thin slice of 2% agarose, and visualized using a confocal microscope (Zeiss LSM780). The experiment was repeated three independent times.

*Flow cytometry.*

We prepared the bacterial cultures as for the confocal microscopy. After that, we took 5.5 μl of washed and concentrated culture and incubated it for 1 h and 30 min (room temperature). Then, we treated the bacteria with 4% Paraformaldehyde (PFA) for 40 min to fix them. We then stained the cells with a DNA marker (Hoechst 33342) for 35 min. After which we diluted the cells in sterile PBS to reach a final volume of 350 µl and processed the samples with a flow cytometer (BD LSRFortessa). For each sample, 50 000 events were measured. We used a variety of controls, such as cells only stained with Hoechst, cells only stained with MitoTracker GreenFM, cells stained with Hoechst + Mitotracker GreenFM and cells without dye to set appropriate gates and to ensure that our analyses were conducted on bacteria that were positive for our green (MitoTracker), red (Cy5 tagged miRNAs) and blue (Hoechst) markers. For each experiment, duplicates for each strain were prepared. The experiment was repeated five independent times for *V. paradoxus* EPS and four times for *B. mycoides* YL123. For our analyses, we used the mean of the duplicates and tested the differences between the strains and treatments with two Kruskal-Wallis tests. As a post-hoc test, we used Dunn’s test for pairwise multiple comparisons (significance=p adjusted < 0.05).

*In vitro miPEP transcriptomic experiment*

*Arabidopsis* culture *in vitro.* Approximately 50 *Arabidopsis thaliana* col-0 seeds were surface-sterilised by soaking them in 70% ethanol for 5 minutes, followed by 20 minutes in 0.5% sodium hypochlorite. Seeds were then rinsed six times with sterile distilled water and resuspended in 1 mL of water, which was poured onto a Petri dish, with some culture medium, to let the water evaporate under sterile conditions. The Petri dish was sealed with parafilm and placed at 4°C for 3 days, for the stratification process, allowing a synchronous germination. The culture medium was composed of 1.2 g of Hoagland’s No. 2 basal salt mixture (Sigma-Aldrich, reference H2395), 8 g of agar ‘suitable for plant cell culture’ (Sigma-Aldrich, ref. A8678) and 500 mL of MilliQ water. The pH of the medium was adjusted with HCl to 5.8-5.9, before adding the final 250 mL of water, resulting in 750 mL of medium. The solution was autoclaved and poured into square petri dishes for plant culture. When hardened, around 2 cm of solid medium was cut out, creating a ridge where the surface-sterilized seeds were positioned (10-15 seeds/dish). Petri dishes with these positioned seeds were sealed with microtape and placed vertically in a growth chamber, with 16 h of daylight at 21°C, 8 h of darkness at 19°C, with 25% relative humidity.

miPEP treatment. Twenty days after the seeds were placed in the growth chamber, the seedlings had developed at least 6 leaves and were ready to be treated. The miPEP treatment consisted in applying 100 µL of a 10 µM solution to each plant, starting at the tip of the root to the base (bottom-to-top). A second treatment was applied 24 h later, before the microbial inoculation. There were three conditions: miPEP159c treatment (MQNLRVHVFLIESARC), a scrambled version of miPEP159c (KHLGRLDRTH) and a water control, each treatment was applied to 7 Petri dishes (biological replicates), containing 10-15 plants each (pseudo-replicates). The use of a scrambled control as well as a solvent control (*i.e.* water) was to ensure that any “miPEP-effect” was indeed due to its action on the plant and not *via* some nutritive or anti-microbial effect of the peptide.

Bacterial growth and inoculation. *Variovorax paradoxus* EPS was first inoculated, from a -20°C stock, on a yeast extract Petri dish (5 g/L) to check that colonies are homogenous. A single colony was transferred into a liquid culture of yeast extract to grow overnight at 30°C and 110 rpm. The overnight culture’s optical density was measured at 600 nm to estimate the number of bacterial cells added to each plant: at OD = 1, the estimated cellular concentration is 3.33 x 10^9^ CFU/mL. 100 µL (with OD = 0.418 *i.e.* ~1.39 x 10^8^ cells) of the overnight culture was directly applied to each plant, from the tip to the base of the root, one hour after the second miPEP treatment.

Sampling. Two hours after the bacterial inoculation, the plants and rhizospheres were sampled. The rhizosphere samples were isolated using a sterile spatula by extracting small squares of medium around the tip of the roots. Whole plants were gently extracted from the medium, by pulling on the aerial system. Samples were flash-frozen in liquid nitrogen and ground shortly after, using a sterile mortar and pestle. The resulting fine powder was stored at -80°C.

RNA extraction. The NucleoSpin ® RNA kit (MACHEREY-NAGEL, Düren, Germany) was used to extract RNA from ~100 mg of previously ground powder, with some changes to the manufacturer’s protocol. During the cell lysis, 400 µL of RA1 solution were used instead of 350µL, followed by 400 µL of ethanol to adjust the RNA binding conditions. The following steps remained the same, up until the elution, where only 30µL of water was used and left to incubate on the column before centrifugation, resulting in better yields. DNase reaction was performed on-column during the protocol.

Target prediction. Potential targets of miRNA159c in the genome of *Variovorax paradoxus* EPS were identified using the online [*psRNAtarget*](https://www.zhaolab.org/psRNATarget/home) tool. On the server, the ath-miRNA159c sequence (UUUGGAUUGAAGGGAGCUCCU), obtained from [miRBase](https://www.mirbase.org/), and the accession number of the EPS genome (CP002417.1) were uploaded for analysis, with the default parameters of the Schema V2 of the 2017 release. Three of the targeted genes were selected for *in vivo* quantification.

RT-qPCR. Retrotranscription was performed using 50 ng of total RNA, more or less diluted to start the reaction with 12 µL of RNA, to which was added 1 µL dNTPs (10 mM) and 1 µL of random primers (200 ng), followed by a 5-minute incubation at 65°C. Samples were then placed on ice for 1min. Sequentially, 4 µL of 5X First-Strand Buffer, 1 µL of DTT (0,1 M) and 1 µL of SuperScript™ Reverse Transcriptase (200 U/µL, Invitrogen) were mixed in by pipetting and the samples incubated for 5 min at 25°C, followed by 1 h at 60°C and 15 min at 70°C. Samples were then placed on ice and 100 µL of RNAse-free water was added to the 25 µL of newly synthesised cDNA. Quantitative PCR was performed using a reaction mix composed of 5 µL of iTaq Universal SYBR Green Supermix (Bio-Rad Laboratories, ref. 1725124), 0.6 µL of primer mix (10 µM, forward & reverse) and 3.4 µL of RNAse-free water, to which 1 µL of cDNA was added. All measurements were performed using three technical replicates, in 384-well plates, on a Roche LightCycler ® 340 thermocycler. The qPCR was programmed as follows : a first step of polymerase activation and DNA denaturation at 95°C for 5 min; then the cycle commenced with further denaturation at 95°C for 10 sec, an annealing step at Tm for 20 sec and an extension step at 72°C for 30 sec; after 40 cycles, a final melting curve was produced: from 65°C to 97°C, with 5 acquisitions per °C. The melting temperature (Tm) for each primer set was adjusted to optimise the amplification specificity and their efficiency was validated beforehand and can be found with primer sequences in Supplementary Table S3. Using a dilution range of a cDNA mixture, each primer set’s efficiency was calculated using the slope of the *x*=log(dilution); *y*=Ct, in the following equation: (10^(-1/(slope))-1)*100, which should be in the 90-110% range. A melting curve was produced at the end of each qPCR program to ensure that a single gene was amplified. In some cases, the amplicon was verified by gel electrophoresis.

Data analysis. After RNA extraction and RT, two samples were discarded from further analysis due to experimental mishaps, resulting in 6 replicates in the “miPEP” and “water” conditions and 7 replicates in the “scrambled” condition. Quantification of pri-miR159c only succeeded in 6 out of 7 “scrambled” replicates and 5 out of 6 “water” replicates. Bacterial genes, *i.e*. Lys R, phosphatidate and alpha-macroglobulin, were quantified relative to RecA and GyrA reference genes, whereas the plant pri-miRNA159c was quantified relative to a plant reference gene, cyclophilin. The number of PCR cycles necessary for the fluorescence to emerge from the background noise is referred to as the “*Ct*”. Noise was automatically determined by the LightCycler 480 software and *Ct*s were calculated with the Abs Quant/2nd Derivative Max - high confidence mode. A coefficient of variation (CV) was calculated for the three technical replicates and a threshold of 2% was applied : extreme technical replicates were excluded, if this limit was exceeded. The remaining technical replicates were averaged to create the *Ct* value for each sample. The analysis of pri-miR159c expression was done using the Pfaffl method, which takes into account the efficiency of each primer set [13]. This method calculates a relative expression ratio based on the difference of *Ct*s of a gene of interest in a sample *versus* a calibrator, and in comparison, with a reference gene. The analysis of bacterial genes relied on two reference genes, which called for a slightly modified version of the Pfaffl method. To calculate a relative expression ratio, we had to normalise our qPCR data using the geometric mean of both reference genes [14]. In both cases, we averaged the samples treated with water to create a calibrator.

Statistical analyses. Statistical analyses were performed on the resulting relative expression ratios, comparing group-to-group, using a Welch Two Sample t-test or a Wilcoxon rank sum exact test, depending on if the data were normal.

**Effect of miRNAs on the bacterial community**

*Arabidopsis mutant experiment*

Plant growth and mutant description. Five *A. thaliana* mutants were grown in a mix of soil and sand (2:1) for a month, in individual pots. Mutants were provided by Hervé Vaucheret and Taline Elmayan (IJPB, INRAE, Versailles, France). *RTL1* mutant over-expresses RTL1 protein which results in a suppression of siRNA pathway without affecting miRNAs [15]. *RTL1myc* over-expresses RTL1 protein flagged with Myc epitope, rendering RTL1 less active, so siRNA pathway is less suppressed than with *RTL1* mutant. *Ago1-27* mutant has AGO protein function partially impaired and is completely post-transcription gene silencing (PTGS) deficient [16]. *Dcl1-2* mutant has total loss of function of DCL1 protein resulting in low levels of miRNA and developmental problems [17]. *Hen1-4* mutant is miRNA defective but is also affected in some siRNA – PTGS [18]. HEN1 methylates siRNA and miRNA to maintain their levels and size, but also to protect them from uridylation and subsequent degradation [19].

DNA extraction, sequencing and qPCR. We grew *A. thaliana* mutants and wild-type plants in quadruplicate for a month and sampled the root, rhizoplane and closely adhering rhizosphere. Two of the wild-type plants were lost during the experiment and subsequent analyses. DNA was extracted using NucleoSpin Plant II kit (Macherey-Nagel). DNA was sent for 16S rRNA gene amplicon (primers 341F: 5’- CCTACGGGNGGCWGCAG- 3’ and 534R: 5’- ATTACCGCGGCTGCTGGCA – 3’) sequencing on a MiSeq system (Illumina) in paired-end mode (2x250 bp) at the Centre d’expertise et de services Génome Québec (Montreal, Canada). In parallel, 16S rRNA gene was quantified by qPCR, using the same primers as for sequencing. Within each well was 0.1 µL of each primer (10 µM), 4 µL LightCycler® 480 SYBR Green I Master (final volume 6 µL PCR mix) and 2 µL of DNA (25 ng.µL^-1^). We used the following 40-cycle program: pre-incubation (95°C- 4 min) / amplification (95°C – 30 s ; 49°C – 1 min ; 72°C – 1 min) / 72°C-10 min/ melting curve (95°C – 5 s ; 49°C – 1 min ; 97°C – continuous 5 measures/ °C) / cooling (40°C – 30 s). At the last step of amplification, a single measurement was performed and then continuously during the melting curve step. A minimum of 3 technical replicates was quantified. The number of copies of 16S rRNA gene was determined in comparison with a standard curve using serial dilutions of plasmids with cloned fragments (R² = 0.994).

Amplicon sequences processing. Amplicon sequencing data was processed with AmpliconTagger [20]. Briefly, contaminants and unpaired reads were removed, and remaining sequences were trimmed to remove adaptors and primers. Reads were then filtered for quality control such that reads having at least one N or having average phred score quality less than 20 were left out. A total of 3,776,292 reads passed the quality control and 1,230,944 sequences were successfully processed to generate ASVs (DADA2) [21]. ASVs were filtered for chimaeras using DADA2’s internal removeBimeraDeNovo (method = ’consensus’) workflow followed by VSEARCH’s [22] UCHIME *de novo*. Each remaining ASV was assigned a taxonomic lineage by using the RDP classifier with the SILVA [23] R138. ASVs assigned to bacterial/archaeal and rendered into ASV abundance tables.

Microbial community analyses. Analyses of ASVs composing the microbial communities were performed using the R package “phyloseq” v 1.32.0 [24]. After checking the rarefaction curves, all samples seemed sufficiently sequenced. For Shannon diversity index calculation, all reads were rarefied to the lowest number of reads found in a sample. Significance of differences in alpha-diversity between mutants was tested using a linear model, whilst checking the normal distribution of residuals and using wild-type (WT) samples as a reference. If residuals did not follow a normal distribution, a general linear model (GLM) with gamma distribution and “log” or “link” scale were used. All tests performed were, by default, two-sided.

Statistical analyses. The effect of miRNA/siRNA mutation in *A. thaliana* on the structure of microbial communities was visualized using Principal Coordinate Analysis (PCoA) ordinations of Bray-Curtis dissimilarities. Permutational Multivariate Analysis of Variance (Permanova) was used to determine the statistical impact of miRNA/siRNA mutations on the microbiota structure, alongside checking group dispersion and pairwise tests.

*miPEP experiment*

Plant growth and miPEP treatment. *Arabidopsis thaliana* Col-0 were sowed on a mix of potting soil and sifted sand (<2.2 mm) (ratio 2:1), in a greenhouse. After germination, seedlings were transferred into individual pots (3 cm diameter, 5 cm depth) that were moved to a growth chamber under 16 h of daylight, 8 h of darkness, at 20°C. When the plants reached the 6-leaf stage, the miPEP treatments started. Plants (16 replicates/condition) were treated with 500 µL of water (control condition) or a miPEP solution (20 µM of miPEP159a: MTWPLLSLSFLLSKYV, miPEP159b: MGLRKVLEMNTIFDSLFLSH or miPEP159c: MQNLRVHVFLIESARC), applied at the base of the crown, 3 times a week for a total of 10 applications. After each application, the trays in the chamber were moved around to diminish any border effect.

Sampling & DNA-RNA extraction. Once the treatments were over, each individual pot was spilled on a sieve and the aerial part was separated from the root system. The roots of two plants were pooled together and briefly rinsed in 10 mL of PBS (1X). The roots and the attached soil were then separated from the rinsing solution, using a funnel and a sterile compress, and were subsequently dried using absorbent paper. The dried roots and rhizosphere were flash frozen in liquid nitrogen and stored at -80°C. DNA was extracted using the NucleoSpin® Plant II mini kit (MACHEREY-NAGEL, Düren, Germany) whereas RNA was isolated with a homemade protocol [25]. The integrity and quantity of the extracted DNA and RNA was estimated using Nanodrop and by running a 1% agarose gel. Amplicon sequencing, processing, qPCR quantification, and analyses were performed as described above.

*Simplified soil community experiment*

Soil microbes enrichment.

The soil microbes were enriched from five different media: 1) Tryptic Soy Broth, 2) Potato Dextrose Broth, 3) Minimal medium + Carbon solution + NH4NO3, 4) Minimal medium + Carbon solution + urea and 5) Minimal medium + Carbon solution + amino acids. The minimal media were supplemented with a solution of artificial carbon-rich root exudates: 20 mM glucose 20 mM fructose, 12 mM sucrose, 20 mM lactic acid, 12 mM citric acid, 16 mM succinic acid. For these media, the final concentration was 0.5 g/L of C and 0.1 g/L of N. To culture the soil microbes, 2 g of sieved agricultural soil (sampled at the Armand-Frappier experimental field: 45.5416N, -73.7173E) was incubated in 20 mL of each media, for 28 h (200 rpm, 25 ºC). The cultures were, filtered 30 µm, normalized to the same optical density, pelleted (4 ºC, 15 min, 4700 *g*), suspended in PBS, pooled and aliquoted into sterile cryotubes. A cryoprotective solution 2x (0.6% (w/v) Tryptic Soy Broth, 10% (v/v) DMSO and 2% (w/v) trehalose) was added to the cultures (1:1 (v/v)) and the cryotubes were gently mixed (5x inverting), left to equilibrate for 20 min and placed at -80 ºC [26]. Cells were revived, inoculated in 5 mL of TSB overnight, washed, normalized to OD600 0.200 and inoculated in a 96-wells plate containing a mixture of 17 amino acids as nitrogen source and artificial root exudates as a carbon source.

Microbial growth and miRNA exposure.

To investigate the potential modification of microbial activity by plant miRNAs, we revived, cultured overnight (in 5 mL of TSB, 28 ºC, 200 rpm), washed (2x) and normalized the soil microbes (OD600 0.200). We then cultured them in a medium containing an equimolar mixture of 17 L-amino acids (15 mM), artificial root exudates (15 mM) and a miRNA treatment that had a final concentration of 10 µM (five biological replicates were prepared). The miRNA treatment consisted of an equimolar mix of five miRNAs (ath-miR158a-3p, ath-miR158b, ath-miR159a, ath-miR827, and ath-miR5642b) of either the plant mimics or scrambled controls (Supplementary Table S2). Microbial activity was quantified by introducing a tetrazolium dye, which undergoes a color change to purple in the presence of dehydrogenases, subsequently intensifying the optical density at 600 nm. Optical density measurements were recorded hourly using a plate reader. The experiment was concluded after 52 hours.

16S rRNA gene amplicon sequencing.

To determine whether alterations in microbial activity were indicative of changes within the bacterial community, we proceeded with a microvolume physical DNA extraction [27] from the cultures and prepared the libraries to sequence the V4-V5 region of the 16S rRNA gene (primers 515F-Y: GTGYCAGCMGCCGCGGTAA and 926R: CCGYCAATTYMTTTRAGTTT) [28] using the MiSeq platform (Illumina) at the Centre d’expertise et de services de Génome Québec, Montreal, Canada. The sequences were processed with the AmpliconTagger pipeline [20], and analysed as described above.

**References**

1. Bolger AM, Lohse M, Usadel B. Trimmomatic: a flexible trimmer for Illumina sequence data. Bioinformatics. 2014;30:2114-2120.

2. Kozomara A, Birgaoanu M, Griffiths-Jones S. miRBase: from microRNA sequences to function. Nucl Acids Res. 2019;47:D155-D162.

3. Li H, Durbin R. Fast and accurate short read alignment with Burrows-Wheeler transform. Bioinformatics. 2009;25:1754-60.

4. Quinlan AR, Hall IM. BEDTools: a flexible suite of utilities for comparing genomic features. Bioinformatics. 2010;26:841-842.

5. Levy A, Salas Gonzalez I, Mittelviefhaus M, Clingenpeel S, Herrera Paredes S, Miao J et al. Genomic features of bacterial adaptation to plants. Nat Genetics. 2017;

6. Dai X, Zhuang Z, Zhao PX. psRNATarget: a plant small RNA target analysis server (2017 release). Nucl Acids Res. 2018;

7. Axtell MJ. Classification and Comparison of Small RNAs from Plants. Ann Rev Plant Biol. 2013;64:137-159.

8. Pearson WR. Finding Protein and Nucleotide Similarities with FASTA. Curr Protoc Bioinformatics. 2016;53:3 9 1-3 9 25.

9. Jones P, Binns D, Chang H-Y, Fraser M, Li W, McAnulla C et al. InterProScan 5: genome-scale protein function classification. Bioinformatics. 2014;30:1236-1240.

10. Manjang K, Tripathi S, Yli-Harja O, Dehmer M, Emmert-Streib F. Graph-based exploitation of gene ontology using GOxploreR for scrutinizing biological significance. Sci Rep. 2020;10:16672.

11. Tremblay J, Schreiber L, Greer C. High resolution shotgun metagenomics the more data the better? Brief Bioinfo. 2022;23:bbac443.

12. Han J-I, Choi H-K, Lee S-W, Orwin PM, Kim J, LaRoe SL et al. Complete genome sequence of the metabolically versatile plant growth-promoting endophyte *Variovorax paradoxus* S110. J Bacteriol. 2011;193:1183-1190.

13. Pfaffl MW. A new mathematical model for relative quantification in real-time RT-PCR. Nucl Acids Res. 2001;29:e45.

14. Vandesompele J, De Preter K, Pattyn F, Poppe B, Van Roy N, De Paepe A et al. Accurate normalization of real-time quantitative RT-PCR data by geometric averaging of multiple internal control genes. Genome Biol. 2002;3:RESEARCH0034.

15. Shamandi N, Zytnicki M, Charbonnel C, Elvira-Matelot E, Bochnakian A, Comella P et al. Plants Encode a General siRNA Suppressor That Is Induced and Suppressed by Viruses. PLoS Biol. 2015;13:e1002326.

16. Morel JB, Godon C, Mourrain P, Béclin C, Boutet S, Feuerbach F et al. Fertile hypomorphic ARGONAUTE (ago1) mutants impaired in post-transcriptional gene silencing and virus resistance. Plant Cell. 2002;14:629-639.

17. Xie Z, Kasschau KD, Carrington JC. Negative feedback regulation of Dicer-Like1 in Arabidopsis by microRNA-guided mRNA degradation. Curr Biol. 2003;13:784-9.

18. Boutet S, Vazquez F, Liu J, Béclin C, Fagard M, Gratias A et al. Arabidopsis HEN1: a genetic link between endogenous miRNA controlling development and siRNA controlling transgene silencing and virus resistance. Curr Biol. 2003;13:843-848.

19. Li J, Yang Z, Yu B, Liu J, Chen X. Methylation protects miRNAs and siRNAs from a 3'-end uridylation activity in Arabidopsis. Curr Biol. 2005;15:1501-7.

20. Tremblay J, Yergeau É. Systematic processing of rRNA gene amplicon sequencing data. GigaScience. 2019;8:giz146.

21. Callahan BJ, McMurdie PJ, Rosen MJ, Han AW, Johnson AJA, Holmes SP. DADA2: high-resolution sample inference from Illumina amplicon data. Nat Meth. 2016;13:581.

22. Rognes T, Flouri T, Nichols B, Quince C, Mahé F. VSEARCH: a versatile open source tool for metagenomics. PeerJ. 2016;4:e2584.

23. Quast C, Pruesse E, Yilmaz P, Gerken J, Schweer T, Yarza P et al. The SILVA ribosomal RNA gene database project: improved data processing and web-based tools. Nucl Acids Res. 2012;41:D590-D596.

24. McMurdie PJ, Holmes S. phyloseq: an R package for reproducible interactive analysis and graphics of microbiome census data. PLoS One. 2013;8:e61217.

25. Griffiths RI, Whiteley AS, O'Donnell AG, Bailey MJ. Rapid method for coextraction of DNA and RNA from natural environments for analysis of ribosomal DNA- and rRNA-based microbial community composition. Appl Environ Microb. 2000;66:5488–5491.

26. Kerckhof FM, Courtens EN, Geirnaert A, Hoefman S, Ho A, Vilchez-Vargas R et al. Optimized cryopreservation of mixed microbial communities for conserved functionality and diversity. PLoS ONE. 2014;9:e99517.

27. Bramucci AR, Focardi A, Rinke C, Hugenholtz P, Tyson GW, Seymour JR et al. Microvolume DNA extraction methods for microscale amplicon and metagenomic studies. ISME Commun. 2021;1:79.

28. Parada AE, Needham DM, Fuhrman JA. Every base matters: assessing small subunit rRNA primers for marine microbiomes with mock communities, time series and global field samples. Environ Microbiol. 2016;18:1403-1414.
